# Oxidative Stress-Induced circHBEGF Promotes Extracellular Matrix Production via Regulating miR-646/EGFR in Human Trabecular Meshwork Cells

**DOI:** 10.1155/2020/4692034

**Published:** 2020-11-24

**Authors:** Wencui Shen, Chengzhi Wang, Bingqing Huang

**Affiliations:** ^1^Tianjin Eye Hospital & Eye Institute, Tianjin Key Laboratory of Ophthalmology and Visual Science, Nankai University, Tianjin 300020, China; ^2^Laboratory of Immunology and Inflammation, Department of Immunology and Research Center of Basic Medical Sciences, Key Laboratory of Immune Microenvironment and Diseases of Educational Ministry of China, Tianjin Key Laboratory of Cellular and Molecular Immunology, Tianjin Medical University, Tianjin 300070, China; ^3^State Key Laboratory of Experimental Hematology, Institute of Hematology and Blood Disease Hospital, Chinese Academy of Medical Sciences and Peking Union Medical College, Tianjin 300020, China

## Abstract

Primary open-angle glaucoma (POAG), a leading cause of irreversible vision loss, presents with increased prevalence and a higher degree of clinical severity in the world. Growing evidence has shown that ncRNAs are involved in the fibrotic process, which is thought to be the proegumenal cause of POAG. Here, we screened out a differentially expressed circRNA (named circHBEGF) in human trabecular meshwork cells (HTMCs) under oxidative stress, which is spliced from pre-HBEGF. circHBEGF promotes the expression of extracellular matrix (ECM) genes (fibronectin and collagen I). Further studies revealed that circHBEGF could competitively bind to miR-646 as a miRNA sponge to regulate EGFR expression in HTMCs. Importantly, HBEGF can also activate EGF signaling pathways, through which can transcriptionally activate ECM genes in HTMCs. In summary, this study investigates the functions and molecular mechanisms of oxidative stress-induced circHBEGF in the regulation of ECM production in HTMCs through the miR646/EGFR pathway. These findings further elucidate the pathogenic mechanism and may identify novel targets for the molecular therapy of POAG.

## 1. Introduction

Glaucoma is a group of optic neuropathic diseases characterized by progressive and permanent blindness caused by retinal ganglion cell (RGCs) degeneration and their axon loss [1]. There are various factors contributing to the pathogenesis of glaucoma, of which oxidative stress is the main cause of IOP elevation in POAG [2, 3]. Under the condition of oxidative stress, much damage to the trabecular meshwork (TM) occur, such as reducing mitochondrial respiratory activity, altering cytoskeletal structures, and destroying cellular DNA [4–6]. Thus, it is necessary to explore and clarify the mechanisms of TM changes caused by oxidative stress.

Both genetic and epigenetic components play important roles in the occurrence and development of POAG [7–9]. In recent years, many studies have focused on the effects of noncoding RNAs on its progression, including microRNAs and lncRNAs [10–13]. Several single-nucleotide polymorphisms (SNPs) of the cyclin-dependent kinase inhibitor 2B antisense noncoding RNA (CDKN2B-AS1) also known as ANRIL have been found and are associated with glaucoma, including rs4977756, rs3217992, and rs2157719 [9, 13–15]. Furthermore, Zhao et al. found that ANRIL can inhibit HTMC apoptosis through the downregulation of miR-7 [11]. In our previous studies, we have revealed that miRNA-483-3p and lncRNA-RP11-820 can regulate ECM production in HTMCs [12, 16]. However, the relationship between circRNAs and the pathogenesis of glaucoma has not been explored.

circRNAs, a novel class of noncoding RNAs, are characterized by covalently closed continuous loop structure [17]. Without the 5′ and 3′ end, circRNAs are difficultly degraded and have permanent stability [18]. Previously, many biological functions of circRNAs have been reported, including cell proliferation, differentiation, apoptosis, fibrosis, and angiogenesis [19–22]. Recently, several circRNAs have been reported with the relationship to ophthalmologic diseases [23, 24]. However, the expression profiles of circles in trabecular meshwork tissues are not well characterized.

In this work, we identified the differential expression profile of circRNAs in HTMCs under oxidative stress by microarray analysis. Of 411 dysregulated circRNAs, 194 circRNAs were upregulated and 217 were downregulated in HTMCs under oxidative stress induction (FC ≥ 2, *p* value < 0.05). The potential function of these circRNAs was calculated by gene ontology (GO) and Kyoto Encyclopedia of Genes and Genomes (KEGG) pathway analysis. We then screened and found that circHBEGF (hsa_circ_0074241) was significantly upregulated under oxidative stress induction. Further studies revealed that circHBEGF can promote ECM production through regulating miR-646/EGFR signaling in HTMCs. The candidate circRNA and its downstream signals may act as novel targets for the treatment of POAG patients.

## 2. Materials and Methods

### 2.1. Human Trabecular Meshwork Cell (HTMCs) Culture

HTMCs were purchased from Sciencell Research Laboratories (San Diego, CA, USA) and were authenticated through responsiveness of myocilin expression after treated by dexamethasone. HTMCs were cultured at 37°C in 5% CO2 in low-glucose Dulbecco's Modified Eagle Medium (DMEM) with L-glutamine, 110 mg/mL sodium pyruvate, 10% fetal bovine serum, 100 *μ*M nonessential amino acids, 100 units/mL penicillin, 100 *μ*g/mL streptomycin sulfate, and 0.25 *μ*g/mL amphotericin B. All reagents were obtained from Gibco (Carlsbad, CA, USA).

To establish an oxidative stress model, HTMCs were treated with 300 *μ*M H_2_O_2_ (Beyotime Ins. Of Bio., Shanghai, China) in serum-free medium for 2 hrs, and then the medium was replaced with serum-free media without H_2_O_2_ cultured for another 2 hrs.

### 2.2. Microarray Analysis

The Agilent-078298 human ceRNA array V1.0 4X180K Microarray (GPL22120; Agilent, Santa Clara, CA, USA) was chosen to screen for Gene expression for HTMCs treated with H_2_O_2_. The raw data were obtained using the Feature Extraction Software 10.7 (Agilent) and normalized using the quantile algorithm with Gene Spring 11.0 (Agilent). The raw data were obtained using the FEATURE EXTRACTION Software 10.7 (Agilent) and normalized using the quantile algorithm with Gene Spring 11.0 (Agilent). The systemic bioinformatic analyses of microarray tests were processed by Novel Bioinformatics Co., Ltd (Shanghai, China). The systemic bioinformatic analysis of the microarray test was processed by Novel Bioinformatics Co., Ltd (Shanghai, China). Briefly, the normalization value was set to 1. The threshold we used to screen upregulated or downregulated circRNAs is fold change ≥ 2.0 and a *p* value ≤ 0.05. The raw microarray data were submitted to the Gene Expression Omnibus database and are available under the accession number GSE126170.

### 2.3. Quantitative Real-Time PCR

Total RNA was isolated using Trizol reagent (Invitrogen, Thermo Fisher, Carlsbad, CA, USA). First-strand cDNA was synthesized from total RNA using M-MLV reverse transcriptase (Invitrogen, Thermo Fisher, Carlsbad, CA, USA) according to the manufacture's instruction. Real-time PCR was performed with FastStart Universal SYBR Green Master (Roche, San Francisco, CA, USA) in 7500 fast Real-Time PCR System (ABI, Carlsbad, CA, USA). Data were expressed as 2^-*ΔΔ*Ct^ values and representative of at least three independent experiments.

For miRNAs, total RNA (3 *μ*g) was reverse transcribed into cDNA using a specific stem-loop primer. Primer sequences of genes used for the amplifications were shown in Table S3.

### 2.4. Lentiviral Expression Vector Generation and Cell Line Selection

To overexpress circHBEGF or EGFR, a DNA fragment carrying circHBEGF or full-length EGFR coding sequence was cloned into the PCDH-CMV-MCS-EF1-Puro lentiviral vector (Addgene, Cambridge, MA, USA) between the EcoRIand BamHI restriction sites. The amplified primers for circHBEGF were as follows: forward primer (5′-CGGAATTCTGCGTGTTGGTCAGGGGTCT-3′) and reverse primer (5′-CGCGGATCCCCATGAATACCCTCAACCCAC-3′).

To express miR-149-5p or miR-646, a DNA fragment carrying pri-miR-149-5p or pri-miR-646 was amplified and cloned into the PCDH-CMV-MCS-EF1-Puro lentiviral vector (Addgene, Cambridge, MA, USA) between the EcoRI and BamHI restriction sites. For cloning the miR-149-5p or miR-646 sponge, briefly, the sponge containing miR-149-5p or miR-646 binding sites was cloned into the BamHI/EcoRI site of the pmiRZip lentiviral vector.

The circHBEGF knockdown oligonucleotide sequences were used as TGACTTGCAAGAGGCAGATCT (sh1) and CTTTTGAGAGTTCTCTCGGCA (sh2). The EGFR knockdown oligonucleotide sequences were obtained from The RNAi Consortium/Public TRC portal (construct ID: TRCN0000195131). The oligonucleotides were annealed and cloned into the AgeI/EcoR1 sites of the shRNA vector pLKO.1-puro (Addgene, Cambridge, MA, USA) according to the instructions. A scrambled sequence was used as a control. The lentiviral particles were packed, and stable HTM cell lines were selected as the previous study.

### 2.5. Western Blot

HTMCs were washed twice in cold PBS. Total protein was extracted using RIPA buffer (150 mM NaCl, 10 mM Tris, pH 7.2, 0.1% SDS,1.0% Triton X-100, 5 mM EDTA, pH 8.0) with 10 × protease inhibitor cocktail (Roche, San Francisco, CA, USA). Total protein extracts (20-100 *μ*g) were separated by 8-12% SDS-PAGE and transferred onto PVDF membrane. Membranes were blocked with 5% nonfat dry milk and incubated overnight with the primary antibodies, antifibronectin, anticollagen I (Abcam, Boston, MA, USA), anti-EGFR, anti-pSTAT3, anti-STAT3, anti-pAKT, anti-AKT, anti-pERK, anti-ERK (Cell Signaling Technology, Boston, MA, USA), and anti-*β*-actin (Santa Cruz Biotechnology, Santa Cruz, CA, USA) at 4°C. After being incubated with secondary antibodies, the antibody-antigen complexes were detected using the Chemiluminescent HRP Substrates (Millipore, Billerica, MA, USA).

### 2.6. Pull-Down Assay with Biotinylated circHBEGF Probe

Pull-down assay was performed as previously described [25]. The biotinylated probe of circHBEGF (sense probe) complementary to the back-splicing junction of circHBEGF was synthesized and dissolved in wash/binding buffer (0.5 M NaCl, 20 mM Tris-HCl, pH 7.5, and 1 mM EDTA). Briefly, 1 × 10^7^ HTMCs were harvested, lysed, and sonicated. The sense or random probes were incubated with streptavidin-coated magnetic beads (Sigma, St Louis, MO, USA) at 25°C for 2 hrs to generate probe-coated magnetic beads. The cell lysates were incubated with probe-coated magnetic beads at 37°C for 4 hrs in a hybridization buffer. Then, the beads were washed with wash buffer, and the RNA complexes bound to the beads were eluted and extracted for RT-PCR analysis. The following probe sequences were used: circHBEGF sense pull-down probe, 5′-GTGCCGAGAGAACTCTCAAAAGGT-3′; and random pull-down probe, 5′-TGATGTCTAGCGCTTGGGCTTTG-3′.

### 2.7. Luciferase Assay

The 3′-UTR of EGFR was amplified by PCR using cDNA from 293T cells and cloned into a p-mirGLO Dual-Luciferase miRNA Target Expression Vector (Promega, Madison, WI, USA). The miR-646 precursor expression vector and p-mirGLO Dual-Luciferase 3′-UTR vector were cotransfected into 293T cells using Lipofectamine 2000 according to the manufacturer's instruction. Cells were harvested and lysed at 48 h after transfection. The interaction between miR-646 and 3′-UTR of EGFR was measured by the Dual-Luciferase assay system (Promega, Madison, WI, USA) as the previous study.

### 2.8. Statistical Analysis

All results were derived from at least three independent experiments. Statistical analysis of data was performed with the Student *t* test using Microsoft Office Excel 2007 software (Redmond, WA, USA). The data were expressed as the mean ± SD using the GraphPad Prism statistical program (San Diego, CA, USA). Differences with *p* < 0.05 were statistically significant.

## 3. Results

### 3.1. circRNA Expression Profiles in Human Trabecular Meshwork Cells under Oxidative Stress

**Figure 1:**
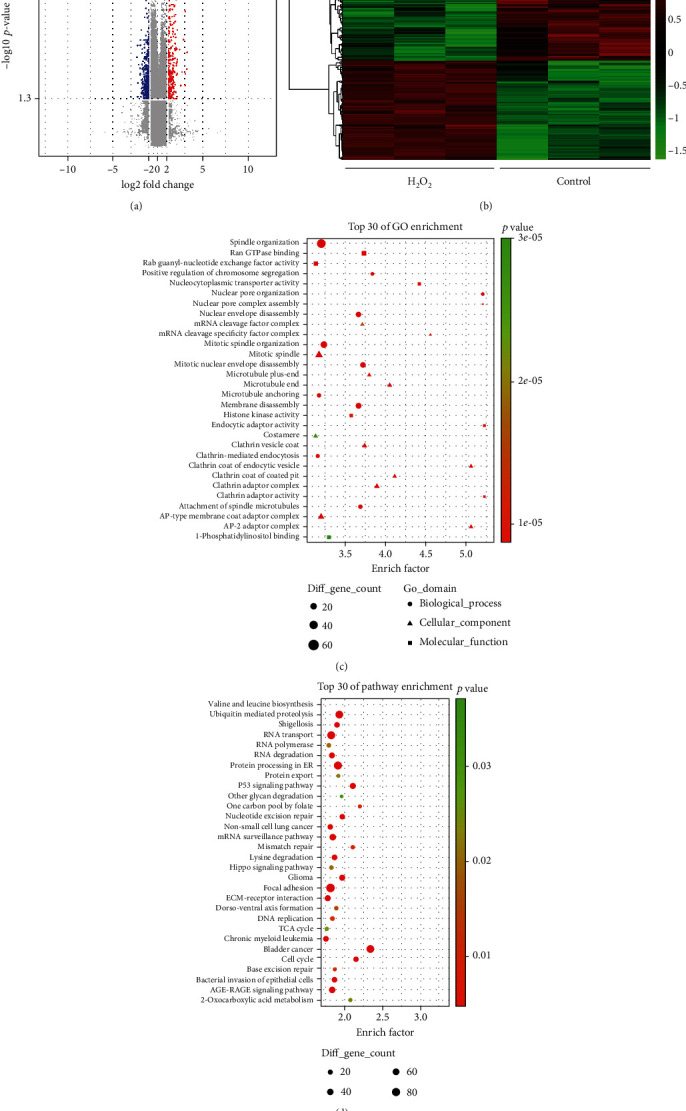
Overview of microarray data of circular RNA (circRNA) expression profiles in HTMCs under oxidative stress condition. (a) Volcano plot is shown of the differentially expressed circRNAs. Red dots represent upregulated circRNAs, and blue dots represent downregulated circRNAs (fold change cut-off: 2.0, p-value cut-off: 0.05). (b) Heat map of cluster analysis showing the different circRNA expression profiles between H_2_O_2_ treated HTMCs and control HTMCs (*n* = 3). (c) Top 30 of GO enrichment analysis of the circRNA host gene. (d) Top 30 of pathway enrichment analysis of circRNA host gene.

Here, we characterized circRNA transcripts by microarray analysis of H_2_O_2_-induced oxidative stress model in HTMCs. In total, 87,525 circRNAs are differentially expressed under oxidative stress, 33,420 circRNAs of which are upregulated, and 54,105 are downregulated. Next, we screened the significantly aberrantly expressed circRNAs (FC ≥ 2.0 and *p* < 0.05) induced by oxidative stress and found that 411 circRNAs were identified as being dysregulated. Among them, 194 were upregulated and 217 were downregulated in HTMCs under oxidative stress (Figure 1(a), Supplementary Table 1). The hierarchical clustering analysis of the differentially expressed circRNAs clearly differentiated circRNAs in HTMCs under oxidative stress condition (Figure 1(b)). We performed GO enrichment and pathway enrichment analysis of those circRNAs to assess their properties and found that focal adhesion and cell cycle signaling of top 30 pathway enrichment were significantly enriched and are possibly related to the roles of circRNAs in HTMCs under oxidative stress (Figures 1(c) and 1(d)). Therefore, oxidative stress-induced circRNAs may play critical roles in regulating ECM production of HTMCs.

### 3.2. circHBEGF Is Upregulated under Oxidative Stress and Promotes Extracellular Matrix Production

**Figure 2:**
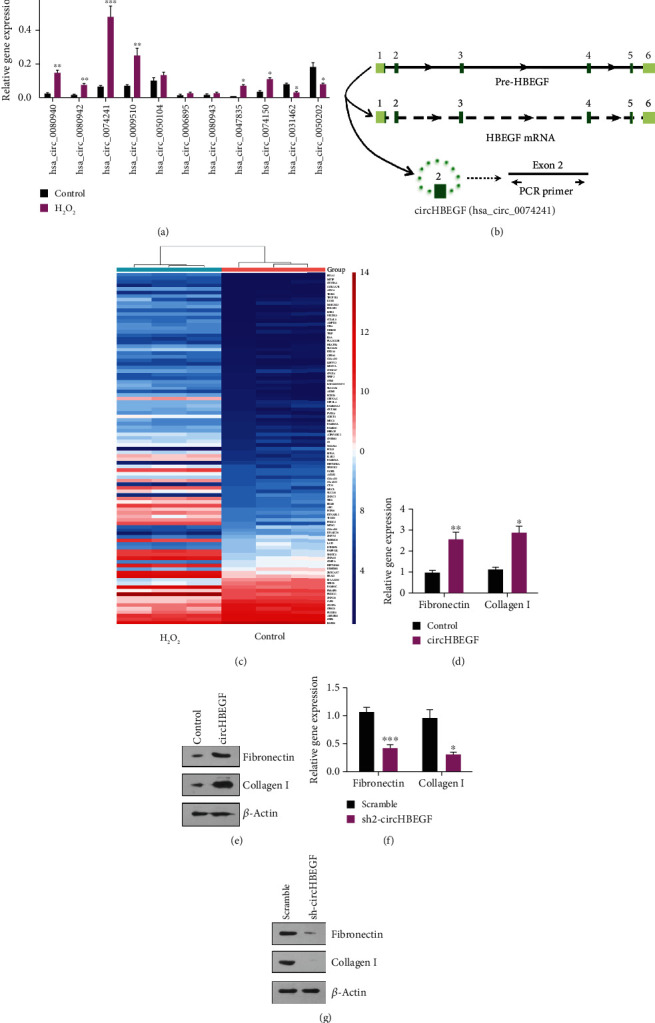
Oxidative stress-induced circHBEGF promotes extracellular matrix genes expression in HTMCs. (a) RT-PCR analysis of 12 screened circRNAs in HTMCs treated with H_2_O_2_ in serum-free medium for 2 hrs, and then the medium was replaced with serum-free media without H_2_O_2_ cultured for another 2 hrs. (b) Pre-HBEGF is the parental gene of circHBEGF. We designed PCR primers to ensure the specificity of circHBEGF detection. (c) Hierarchical clustering of the top 100 differentially regulated protein-coding gene expression data in HTMCs treated with H_2_O_2_ (300 vs. 0 *μ*M). (d) RT-PCR analysis of ECM genes (fibronectin and collagen I) in HTMCs stably expressing circHBEGF and control cells. (e) Western blot analysis of ECM genes in HTMCs stably expressing circHBEGF and control cells. (f) RT-PCR analysis of ECM genes in circHBEGF-knockdown HTMCs and scramble cells. (g) Western blot analysis of ECM genes in circHBEGF-knockdown HTMCs and scramble cells. The values represent the mean ± SD of three independent experiments performed in triplicate. ^∗^*p* < 0.05; ^∗∗^*p* < 0.01; ^∗∗∗^*p* < 0.001.

We next selected top 11 differentially expressed circRNAs from profiles and validated their expression levels under oxidative stress condition. Among selected circRNAs, we found that the expression level of hsa_circ_0074241 was intensely higher under oxidative stress (Figure 2(a)). hsa_circ_0074241 was shown to be a circRNA formed by cyclization of exon 2 of pre-HBEGF (Figure 2(b)). Therefore, we named this circRNA as circHBEGF. A linear HBEGF mRNA was also generated during the formation of circHBEGF (UCSC Genome Browser) (Supplementary Figure 1a). Interestingly, we found that the expression level of HBEGF was significantly higher in HTMCs under oxidative stress from microarray analysis (Figure 2(c)).

To investigate the biological roles of circHBEGF in ECM production, we stably expressed circHBEGF in HTMCs using a lentivirus delivery system (Supplementary Figure 1b) and found that HTMCs stably expressing circHBEGF significantly upregulated fibronectin and collagen I expression (Figures 2(d) and 2(e)). Consistent with the ectopic study, the inhibition of circHBEGF in HTMCs noticeably decreased the mRNA and protein level of fibronectin and collagen I (Figures 2(f) and 2(g)). Taken together, these results indicate that oxidative stress-induced circHBEGF can promote extracellular matrix production in HTMCs.

### 3.3. circHBEGF Directly Sponges miR-646

**Figure 3:**
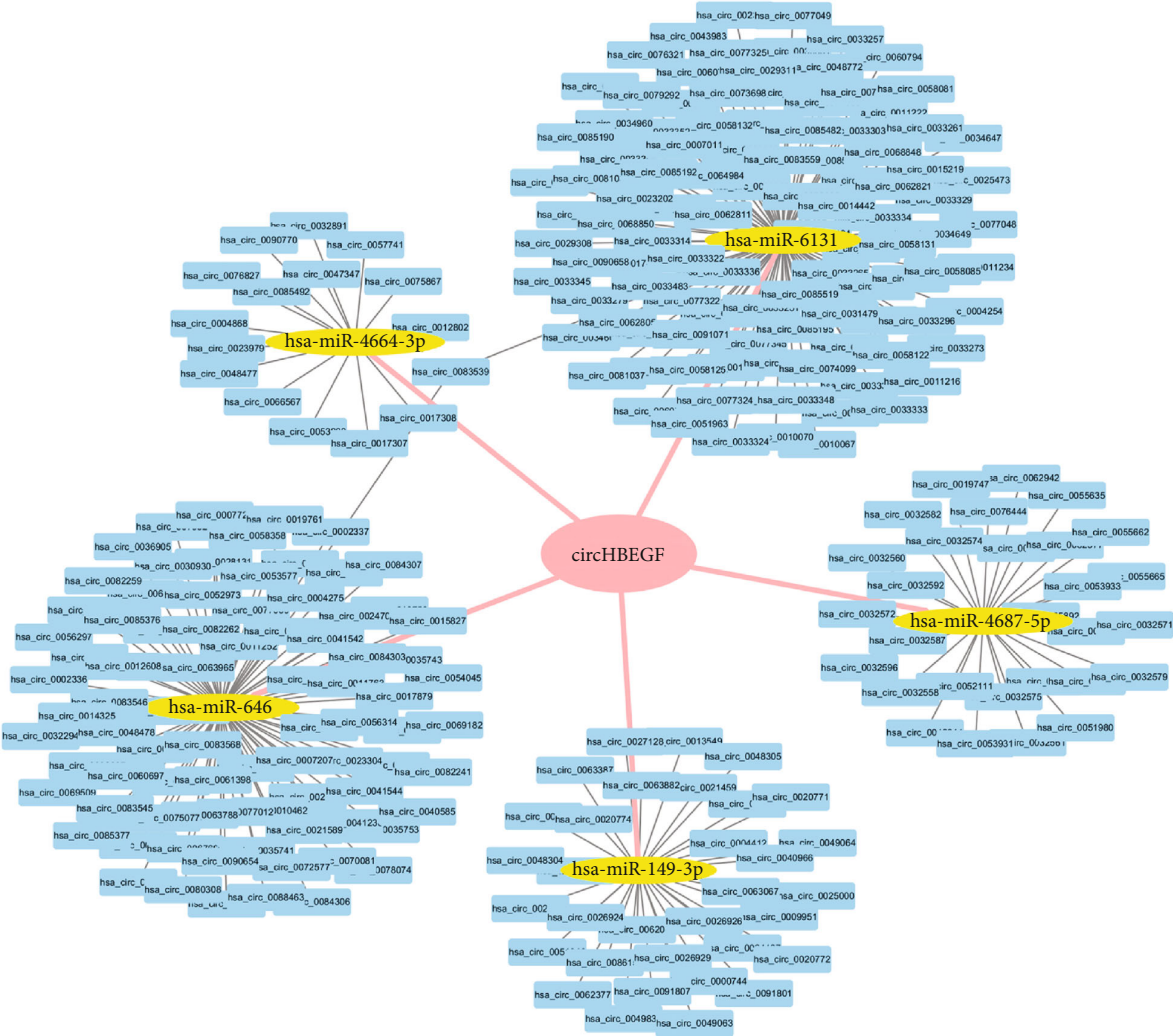
The interaction network between circRNAs and miRNAs in HTMCs. **c**ircHBEGF probably sponge up miR-149-5p, miR-646, miR-4664-3p, miR-4687-5p, and miR-6131 in HTMCs.

Recent studies have revealed that circRNA mainly function as a ceRNA that binds to miRNA [28, 29]. From microarray analysis, we found that circHBEGF most likely binds to miR-149-5p, miR-646, miR-4664-3p, miR-4687-5p, and miR-6131 (Figure 3).

**Figure 4:**
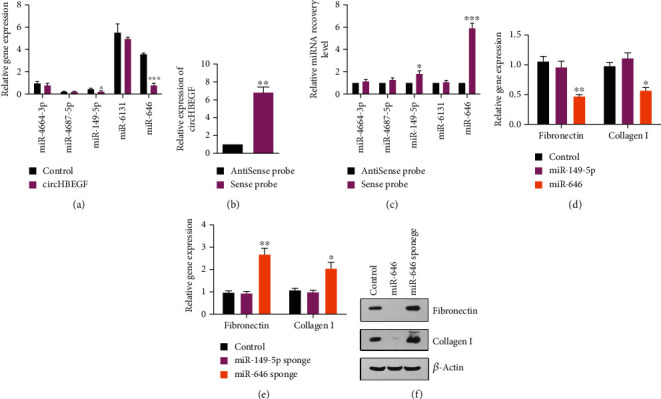
circHBEGF interacts with miR-646. (a) RT-PCR analysis of miR-149-5p, miR-646, miR-4664-3p, miR-4687-5p, and miR-6131 in HTMCs stably expressing circHBEGF and control cells. (b) Lysates from HTMCs were prepared and subjected to RNA pull-down assay with the indicated probe. The expression of circHBEGF was tested by RT-PCR. (c) RT-PCR analysis of 5 miRNA candidates in lysate HTMCs after RNA pull-down with the indicated probe. (d) RT-PCR analysis of ECM genes in HTMCs stably expressing miR-149-5p or miR-646. (e) RT-PCR analysis of ECM genes in HTMCs stably expressing miR-149-5p sponge or miR-646 sponge. (f) Western blot analysis of ECM genes in control HTMCs and miR-646–overexpressing cells or miR-646 sponge expressing cells. The values represent the mean ± SD of three independent experiments performed in triplicate. ^∗^*p* < 0.05; ^∗∗^*p* < 0.01; ^∗∗∗^*p* < 0.001.

To explore their relationship, we detect these five miRNA levels after stably overexpressing circHBEGF and found that circHBEGF can decrease miR-149-5p and miR-646 levels, basically only the expression of miR-646 seems to be affected (about fourfold) (Figure 4(a)). To further determine the functional miRNA targets of circHBEGF, we performed a circRNA pull-down assay with a circHBEGF-specific probe (Supplementary Figure 2a). As a result, circHBEGF and miR-646 were significantly enriched by the circHBEGF-specific probe, slightly as well as miR-149-5p (Figures 4(b) and 4(c)). We then questioned whether miR-646 or miR-149-5p could affect ECM production of HTMCs. We stably expressed miR-646 or miR-149-5p using a lentiviral delivery system in HTMCs (Supplementary Figure 2b) and found that HTMCs stably expressing miR-646 had a decreased level of ECM genes, not for miR-149-5p (Figure 4(d)). We also constructed a lentiviral vector expressing a “sponge” to adsorb miR-646 or miR-149-5p (Supplementary Figure 2c) and found that the inhibition of miR-646 in HTMCs led to an increased expression level of ECM (Figure 4(e)). Western blot results were consistent with the mRNA expression of ECM genes in HTMCs (Figure 4(f)). Taken together, these results indicate that circHBEGF upregulates ECM genes through miR-646.

### 3.4. miR-646 Directly Targets EGFR

**Figure 5:**
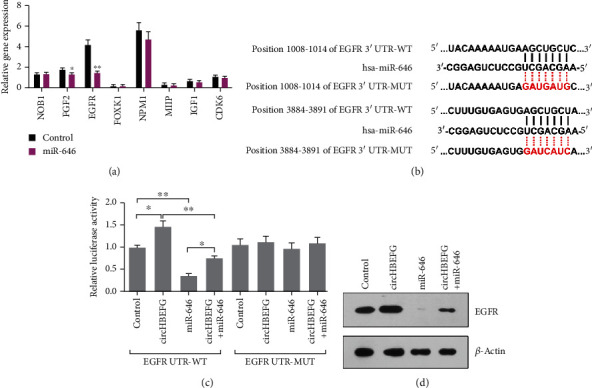
miR-646 targets EGFR. (a) RT-PCR analysis of candidate genes in HTMCs stably expressing miR-646. (b) Sequences of miR-646 and the potential miR-646 binding site of 3′ UTR of EGFR. Also shown are nucleotides mutated in EGFR 3′ UTR-MUT. (c) Dual-Luciferase assays showing the circHBEGF and miR-646 regulation of wild-type EGFR UTR (EGFR UTR-WT) or mutant EGFR UTR (EGFR UTR-MUT). (d) Western blot analysis of EGFR in control cells and circHBEGF or miR-646-overexpressing cells. The values represent the mean ± SD of three independent experiments performed in triplicate. ^∗^*p* < 0.05; ^∗∗^*p* < 0.01.

Having observed that miR-646 could inhibit ECM production in HTMCs, we next planned to find out the target for miR-646. To identify the downstream targets of miR-646, we used mRNA target-predicting algorithms (TargetScan and TargetRank) based on the presence of binding sites in the 3′ UTR and the mRNA expression profile under oxidative stress. Of the eight selected genes that are associated with cell proliferation, migration, adhesion, and invasion, we found that miR-646 could notably reduce the expression level of EGFR (Figure 5(a)). Evaluation of the 3′ UTR sequence of EGFR revealed two binding sites with perfect match sequences for miR-646 (Figure 5(b)).

To further confirm a direct relationship between miR-646 and EGFR, we cloned the 3′ UTR of EGFR into a Dual-Luciferase UTR vector. Luciferase assay results revealed that miR-646 significantly suppressed the 3′ UTR of EGFR, but circHBEGF led to an increased luciferase activity (Figure 5(c)). In addition, circHBEGF could offset the inhibition of EGFR induced by miR-646 (Figure 5(c)). We next generated mutations in the binding site to abrogate the miR-646-EGFR 3′ UTR interaction (Figure 5(b)) and found that the EGFR 3′ UTR carrying mutated binding sites was resistant to regulate by miR-646 and circHBEGF (Figure 5(c)). As expected, there was a positive correlation between the protein level of EGFR and circHBEGF, but a negative correlation with miR-646 in HTMCs (Figure 5(d)). Taken together, these results indicate that miR-646 directly target EGFR in HTMCs.

### 3.5. circHBEGF Promotes Extracellular Matrix Production through Regulating EGFR Signaling Pathways

**Figure 6:**
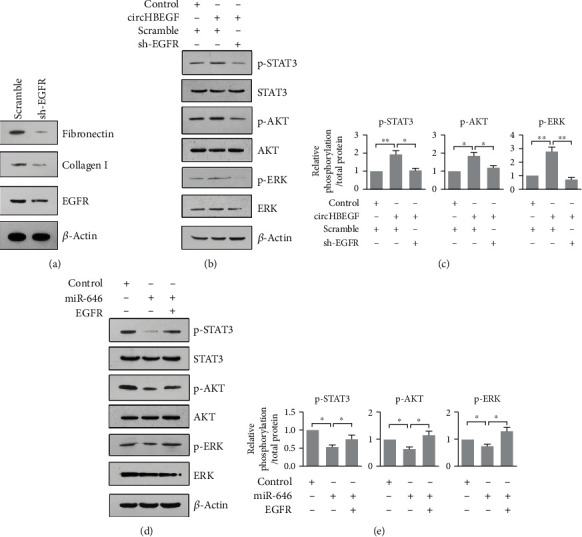
circHBEGF promotes ECM genes through EGF signaling. (a) Western blot analysis of EGFR and ECM genes in HTMCs after EGFR knockdown. (b, d) Western blot analysis of p-STAT3, p-AKT and p-ERK, in HTMCs transfected with the indicated condition. (c, e) Quantification of p-STAT3, p-AKT, and p-ERK related to total protein in (b) and (d).

To investigate the effect of EGFR on ECM genes expression, we stably expressed sh-RNA targeted EGFR in HTMCs and found that knockdown of EGFR in HTMCs led to a decreased expression level of ECM genes (Figure 6(a)). Downstream signaling pathways of EGFR included STAT3, AKT, and ERK [28–30]. In our previous studies, we have revealed that SMAD3 and STAT3 participated in transcriptionally regulating ECM genes expression in HTMCs [12, 16]. To further explore whether circHBEGF or miR-646 can influence activities of STAT3, AKT, and ERK, we stably expressed circHBEGF or miR-646 in HTMCs and found that circHBEGF notably raised the phosphorylation level of STAT3, AKT, and ERK, while miR-646 had opposite effects (Figures 6(b)–6(e)). Importantly, the knockdown or overexpression of EGFR can neutralize the effects of circHBEGF or miR-646 on STAT3, AKT, and ERK in HTMCs (Figures 6(b)–6(e)). All in all, the results further revealed that circHBEGF promotes ECM production through EGFR signaling pathways in HTMCs.

## 4. Discussion

Glaucoma still represents the leading cause of blindness worldwide. Although the morbidity becomes higher, the mechanism of POAG has not been elaborated entirely [31]. circRNAs, a newly recognized kind of ncRNA, has been reported to play a role in regulating both health and disease. To investigate the specific effect of circRNAs in POAG, we have performed a microarray analysis of HTMC under oxidative stress. And we chose circHBEGF for further study through a series of comprehensive data analysis.

**Figure 7:**
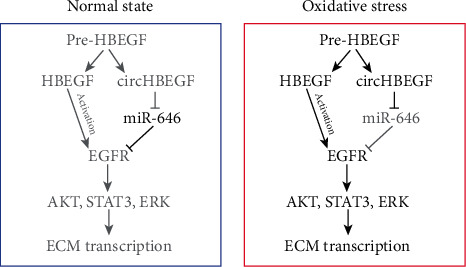
Proposed working model based on our studies. Schematic summarizing our proposed model for circHBEGF in promoting ECM production in HTMCs. circHBEGF directly adsorb miR-646 as a sponge, through which regulate the expression of EGFR. Therefore, circHBEGF can activate EGF signaling (STAT3, AKT, and ERK), through which transcriptionally upregulates ECM gene expression in HTMCs.

In our study, we showed that circRNA expression profiles are significantly different in HTMCs under oxidative stress compared with the normal ones, including 194 upregulated and 217 downregulated circRNAs. Of the 411 differentially expressed circRNAs, we found that the relative expression of circHBEGF was significantly increased in HTMCs under oxidative stress. High circHBEGF expression serves as a sponge of miR-646 and was correlated with ECM accumulation. Further studies revealed that miR-646 directly binds with the 3′ UTR of EGFR. This study reveals how circHBEGF regulates miR-646/EGFR/ECM axis in HTMCs (Figure 7).

At present, some studies have identified numerous genes of differentially expressed circRNAs during the fibrotic process. A recent study showed 67 aberrantly expressed circRNAs in idiopathic pulmonary fibrosis (IPF) patients, the majority of which were generated from exonic regions [32]. Song and colleagues validated intervertebral disc degeneration- (IDD-) specific circRNAs profiles, and they verified that circRNA_104670 is significantly upregulated in human IDD tissues [33]. Therefore, abnormal-expressed circRNAs may participate in the fibrosis of the pathological state. Moreover, a close interaction among lncRNAs/circRNAs and miRNAs has been reported earlier, and both lncRNAs and circRNAs can act as the sponges of miRNAs, competitively suppressing miRNA activity [34, 35]. In a study of human hypertrophic scars (HS), a ceRNA network was built, which can indicate the lncRNA-miRNA-circRNA-mRNA associations [36]. While, in human cartilage degradation, Liu and his colleagues found several miRNA-binding sites of circRNA-MSR, further explaining the role of circRNA-MSR in chondrocyte ECM degradation. So, circRNA may produce the regulatory effect through silencing miRNA in ECM accumulation. In the present study, we also predicted the potential binding miRNAs of circHBEGF and successfully confirmed the targeting relationship between circHBEGF and miR-646.

To investigate how miR-646 exerts its regulatory effects on ECM transcription, we used microRNA target databases and found EGFR was one of its potential targets. EGFR, a transmembrane glycoprotein, is a member of the tyrosine kinase receptor family. When the downstream signaling pathways are activated, specific biochemical reactions occur, like phosphorylation [37, 38]. Some researchers have recognized that the EGER signaling pathway is overactivated during renal fibrogenesis [39, 40]. And some kinase inhibitors of EGER signaling, like Gefitinib, have been verified to decrease lung fibrosis [41]. Besides the small molecule inhibitors, the circRNA/miRNA pathway has also been reported to regulate the EGFR pathway in some disease models. In nasopharyngeal carcinoma, hsa_circRNA-006660 was confirmed to target EGFR through the modulation of miR-1276 [42]. In our work, we found that circHBEGF/miR-646 had a similar effect on targeting EGFR.

To further explore the mechanism of EGFR and the downstream signaling in ECM regulation, we observed the activation of STAT3, AKT, and ERK after the overexpression of circHBEGF or miR-646. In our previous study, we have recognized the role of STAT3 in accelerating the ECM component production of HTMCs [12]. Seo and colleagues focused their attention on STAT3 signaling in renal fibrosis, and they have found Fyn, a member of the Src family of kinases, could be the upstream activator of STAT3 [43]. And the phosphorylation of ERK was reported earlier by EGFR activation [44]. A recent study has shown the regulatory mechanism of EGFR/ERK/AKT signaling in colorectal cancer cell growth and migration [45]. We have found a similar mechanism of EGFR/STAT3/AKT/ERK pathway in regulating ECM transcription of HTMCs, as a target of circHBEGF/miR-646.

In conclusion, the abnormal expression profile of circRNAs and mRNAs of HTMCs induced by oxidative stress, which could provide some pathogenetic factors of POAG, was constructed. Particularly, we observed the promotion effect of circHBEGF on ECM production by directly targeting miR-646. Furthermore, the downstream pathway EFGR/AKT/STAT3/ERK participates in ECM transcription of HTMCs. Above all, more details of pathogenic mechanisms were explained for POAG, which may reveal new potential targets of POAG.

## Data Availability

The NCBI Gene Expression Omnibus accession number for the microarray data reported in this paper is GSE126170.

## Abbreviations

circRNA: Circular RNA
ECM: Extracellular matrix
HBEGF: Heparin-binding EGF like growth factor
POAG: Primary open-angle glaucoma
TM: Trabecular meshwork
HTMCs: Human trabecular meshwork cells
IOP: Intraocular pressure
AH: Aqueous humor
JCT: Juxtacanalicular connective tissue
(SNPs): Single-nucleotide polymorphisms
Fold change: FC

## Supplementary Materials

Supplementary 1 Supplementary Figure S1: related to Figure 2. (a) Transcript information of HBEGF annotated in the UCSC Genome Browser. (b) RT-PCR analysis of circHBEGF in HTMCs stably expressing circHBEGF. (c) RT-PCR analysis of circHBEGF and HBEGF in HTMCs stably expressing shRNA-circHBEGF. ^∗^*p* < 0.05; ^∗∗^*p* < 0.01.

Supplementary 2 Supplementary Figure S2: related to Figure 4. (a) Graphic location of biotinylated circHBEGF probe. (b) RT-PCR analysis of miR-149-5p and miR-646 in HTMCs stably expressing pri-miR-149-5p or pri-miR-646. (c) RT-PCR analysis of miR-149-5p and miR-646 in HTMCs stably expressing miR-149-5p or miR-646 sponge. ^∗∗^*p* < 0.01; ^∗∗∗^*p* < 0.001.

Supplementary 3 Table S1: differentially expressed circRNAs.

Supplementary 4 Table S2: differentially expressed mRNAs.

Supplementary 5 Table S3: the sequences of the RT-PCR primers (5′-3′).
