# Supplementary figures and images for: Oxidative Stress-Induced circHBEGF Promotes Extracellular Matrix Production via Regulating miR-646/EGFR in Human Trabecular Meshwork Cells
Source: Oxid Med Cell Longev. 2020 Nov 24;2020:4692034. doi: 10.1155/2020/4692034 (PMC7722639; doi:10.1155/2020/4692034)

**A**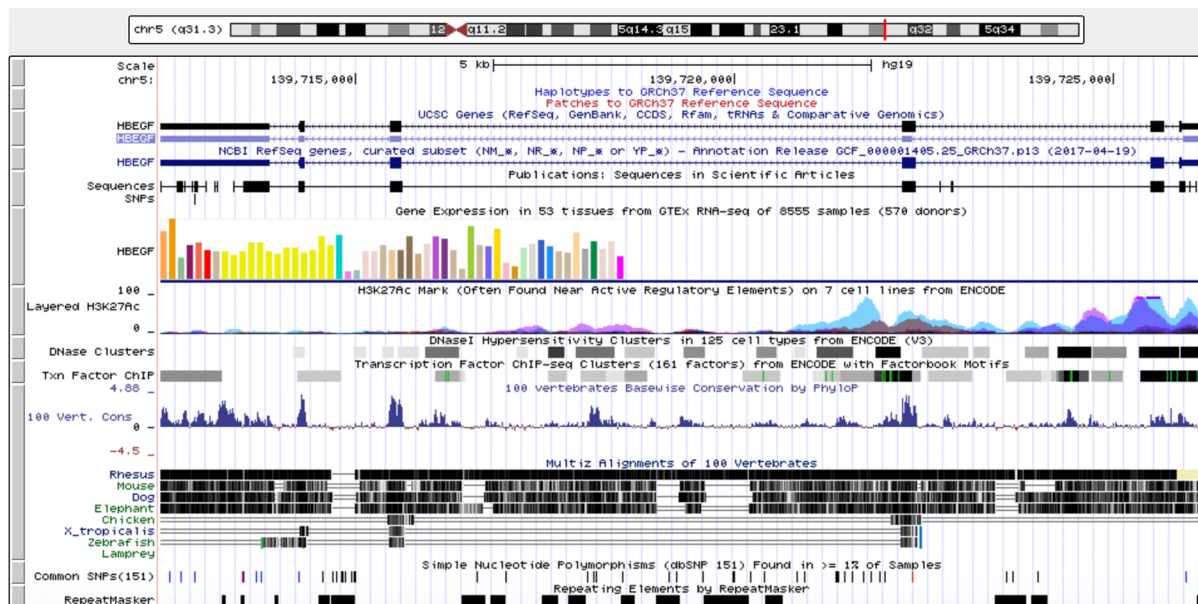**B**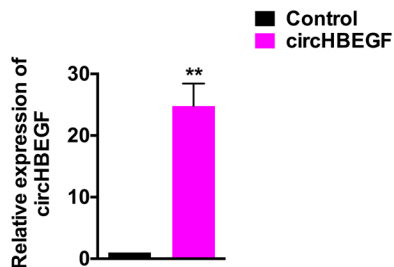**C**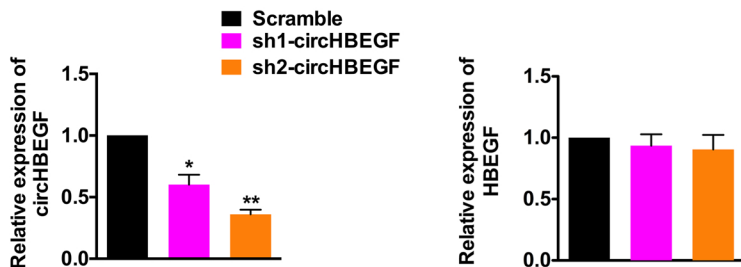

Supplement: Supplementary 1 — Supplementary Figure S1: related to Figure 2. (a) Transcript information of HBEGF annotated in the UCSC Genome Browser. (b) RT-PCR analysis of circHBEGF in HTMCs stably expressing circHBEGF. (c) RT-PCR analysis of circHBEGF and HBEGF in HTMCs stably expressing shRNA-circHBEGF. ∗p < 0.05; ∗∗p < 0.01. [file 4692034.f1.pdf]

**A**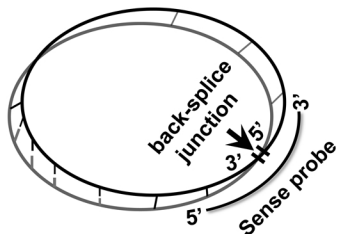**B**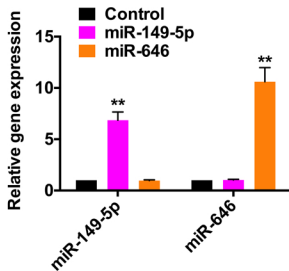**C**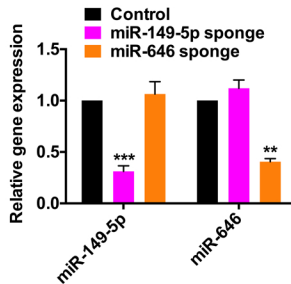

Supplement: Supplementary 2 — Supplementary Figure S2: related to Figure 4. (a) Graphic location of biotinylated circHBEGF probe. (b) RT-PCR analysis of miR-149-5p and miR-646 in HTMCs stably expressing pri-miR-149-5p or pri-miR-646. (c) RT-PCR analysis of miR-149-5p and miR-646 in HTMCs stably expressing miR-149-5p or miR-646 sponge. ∗∗p < 0.01; ∗∗∗p < 0.001. [file 4692034.f2.pdf]
