# Supplementary material for: Oxidative Stress-Induced circHBEGF Promotes Extracellular Matrix Production via Regulating miR-646/EGFR in Human Trabecular Meshwork Cells
Source: Oxid Med Cell Longev. 2020 Nov 24;2020:4692034. doi: 10.1155/2020/4692034 (PMC7722639; doi:10.1155/2020/4692034)
Supplement: Supplementary 3 — Table S1: differentially expressed circRNAs. [file 4692034.f3.pdf]

Column A: Title ;
Column B: Mean ;
Column C: Example;

Title	Mean	Example
Title	Mean	Example
ProbeName	Probe name	hsa_circ_0000026
ControlType	quality control site（pos:Quality Control Point，FALSE: Non-Quality Control Point）	Pos/FALSE
Sample_NS	Normalized signal values of probes in samples	7.023509
Sample_flag	Detection and determination of samples：A：There is no significant difference between the signal of the probe and background; P：There is significant difference between the signal of the probe and background; M：The difference between the signal of the probe and background is between A and P.	P/M/A
Fold_change(1_vs_2)	（formula : foldchange=average(power(2,signal(CASE)))/ average(power(2,signal(CONTROL)))）Multiple differences of two groups (Biologically repetitive samples) or two (No biologically duplicated samples)	3.4
foldchange (abs)	Absolute value of multiple differences of two groups (Biologically repetitive samples) or two (No biologically duplicated samples)	3.4
Regulation(1_vs_2)	Up-down trend of two groups (Biologically repetitive samples) or two (No biologically duplicated samples)	up/down
Type	RNAtype：circRNA/lncRNA / mRNA	circRNA/lncRNA/mRNA
ProbeSeq	Probe sequence	AAGGTATCTTAAGGAAAGTTC AAGGTAAAAAGACAGAGGTGT TTATGGTTACATGTGTGT
circRNA_ID	Gene ID in circbase database	hsa_circ_0082053
chrom	circRNA derived chromosome	Chr7
Hsa_hg19_circRNA_start	Sequence starting position	91787463
Hsa_hg19_circRNA_end	Sequence termination position	91810001
Hsa_hg19_circRNA_strand	Sources of positive and negative chain of circRNA sequence	+/-
genomic_length	Sequence length on genome	53243
spliced_seq_length	Sequence length after splicing	2700
best_transcript	Best transcript	NM_024913
gene_symbol	Source parent gene name	CCDC88C

Fold-Change cut-off: 2.0
P-value cut-off: 0.05
compare: g2_VS_g1
foldchange: the ratio (not logarithm) of normalized intensities between two conditions; Formula: foldchange =average(power(2,signal(CASE)))/average(power(2,signal(CONTROL))).
foldchange (abs): the absolute ratio (not logarithm) of normalized intensities between two conditions; Formula: if foldchange >1,foldchange (abs)=foldchange ,foldchange <1,foldchange (abs)= 1/foldchange .

Probe ID	T test p-value	Fold Change			Normalized signal (log2) of g2			Detection of g2		
ProbeName	pvalues	foldchange	foldchange (abs)	Regulation	1_HTMC_H2O2_	2_HTMC_H2O2_	3_HTMC_H2O2_	1_HTMC_H2O2_fl	2_HTMC_H2O2_fl	3_HTMC_H2O2_fl
hsa_circ_0019389	0.001306124	2.348328498	2.348328498	up	5.99946866	6.158043539	5.824024594	P	P	P
hsa_circ_0034233	0.002055706	0.492254038	2.031471399	down	6.908623193	7.138336658	7.013139329	P	P	P
hsa_circ_0024013	0.028933898	0.494820054	2.020936684	down	5.087367507	4.831146506	4.955491052	P	P	P
hsa_circ_0015764	0.003221368	0.392839018	2.545571987	down	6.339518382	6.537480017	6.376067926	P	P	P
hsa_circ_0011134	0.004551023	4.056031348	4.056031348	up	12.80461416	12.3953919	12.6648352	P	P	P
hsa_circ_0034042	0.000326759	0.479503736	2.085489487	down	5.168363241	5.296386617	5.322481443	P	P	P
hsa_circ_0023852	0.00264189	2.864965138	2.864965138	up	10.10816594	10.15277973	10.16525918	P	P	P
hsa_circ_0035807	0.005974209	0.367676347	2.719783332	down	4.404616153	4.293310273	3.740922345	P	A	A
hsa_circ_0084230	0.001448393	0.455480885	2.195481813	down	5.810586372	6.132592745	5.99190368	P	P	P
hsa_circ_0027493	4.79632E-05	2.253332998	2.253332998	up	11.27781984	11.35057681	11.33355138	P	P	P
hsa_circ_0035768	0.04560067	0.412746787	2.422792934	down	4.758870516	4.441212329	4.33148763	P	A	A
hsa_circ_0082477	0.005525715	0.382141707	2.616830309	down	8.965964658	8.904693221	8.956192257	P	P	P
hsa_circ_0006417	0.021153462	0.361209084	2.768479655	down	8.161517138	8.068832367	8.536767399	P	P	P
hsa_circ_0010126	0.004858443	2.014862579	2.014862579	up	6.383866003	6.520955735	6.737928249	P	P	P
hsa_circ_0055559	0.013537782	2.882106994	2.882106994	up	6.128597196	6.259795291	6.212112037	P	P	P
hsa_circ_0035780	0.010021903	0.451608601	2.21430681	down	4.326288297	4.767488663	4.927842818	P	P	P
hsa_circ_0034646	0.000225472	3.577380813	3.577380813	up	11.75786111	11.84989602	11.77251865	P	P	P
hsa_circ_0002677	0.000252325	3.249513441	3.249513441	up	9.482811116	9.501176412	9.32269605	P	P	P
hsa_circ_0081285	0.016428432	0.49960742	2.001571553	down	5.334308091	4.863543042	5.21669413	P	P	P
hsa_circ_0023854	2.81771E-05	3.232009247	3.232009247	up	10.45576198	10.50941511	10.60372385	P	P	P
hsa_circ_0055279	0.02593077	2.053789548	2.053789548	up	7.792276666	7.52979037	7.832098529	P	P	P
hsa_circ_0015420	3.29596E-05	3.889989923	3.889989923	up	8.866751303	8.73147523	8.889116455	P	P	P
hsa_circ_0051796	0.007416318	2.110501475	2.110501475	up	8.270708178	8.202129702	8.080081383	P	P	P
hsa_circ_0005057	0.003869266	2.896488391	2.896488391	up	7.570347053	7.726243936	7.66004202	P	P	P
hsa_circ_0016695	0.004045757	2.075881553	2.075881553	up	6.328280671	6.597482905	6.56710057	P	P	P
hsa_circ_0022568	0.028747147	2.009929394	2.009929394	up	8.096300776	7.840353181	7.813340835	P	P	P
hsa_circ_0064557	0.002524815	0.351295521	2.846606176	down	3.504930973	3.959192405	3.57022147	A	A	A
hsa_circ_0002830	0.000440065	0.499345698	2.002620638	down	5.162164508	5.234566464	5.146708098	P	P	P
hsa_circ_0017031	0.031941669	0.420450714	2.378400051	down	4.414176697	4.751825831	4.318817901	P	P	A
hsa_circ_0017084	0.002507034	0.452616216	2.209377316	down	5.119330721	5.049692331	5.281031081	P	P	P
hsa_circ_0085102	0.004161228	0.485700408	2.058882353	down	5.069190448	5.264834022	4.850960069	P	P	P
hsa_circ_0057623	0.029015285	0.471605445	2.12041657	down	4.146813775	4.381351214	3.98562232	A	A	A
hsa_circ_0035745	0.036824282	0.48994872	2.041029926	down	5.072694223	4.852867623	4.294919181	P	P	A
[illegible]	[illegible]	[illegible]	[illegible]	[illegible]	[illegible]	[illegible]	[illegible]	[illegible]	[illegible]	[illegible]

Probe ID	Normalized signal (log2) of g1			Detection of g1			Annotation		
ProbeName	1_HTMC_no_NS	2_HTMC_no_NS	3_HTMC_no_NS	1_HTMC_no_flag	2_HTMC_no_flag	3_HTMC_no_flag	ProbeName.1	Type	ProbeSeq
hsa_circ_0019389	4.683796466	4.750339771	4.865874781	P	P	P	hsa_circ_0019389	circRNA	TCTACAAGAGAT
hsa_circ_0034233	7.83657588	8.155851164	8.123531013	P	P	P	hsa_circ_0034233	circRNA	TTACTCAGAGTTC
hsa_circ_0024013	6.355671347	5.754570653	5.729509447	P	P	P	hsa_circ_0024013	circRNA	TTATTCGAGGAT
hsa_circ_0015764	7.608583311	8.014561356	7.645828448	P	P	P	hsa_circ_0015764	circRNA	TTTGATCATGGA
hsa_circ_0011134	11.03163746	10.42501951	10.25849674	P	P	P	hsa_circ_0011134	circRNA	TGAGAAATAAAC
hsa_circ_0034042	6.254286143	6.451628169	6.258192573	P	P	P	hsa_circ_0034042	circRNA	ACAACAACATTC
hsa_circ_0023852	8.457976441	8.626006787	8.770406657	P	P	P	hsa_circ_0023852	circRNA	TAAGGCACAGTT
hsa_circ_0035807	5.454904351	5.425603088	5.917733996	P	P	P	hsa_circ_0035807	circRNA	CTTCATCAAGGT
hsa_circ_0084230	6.913414438	7.138131322	7.281500056	P	P	P	hsa_circ_0084230	circRNA	TTCCAAAAGTGG
hsa_circ_0027493	10.14388029	10.08801793	10.21218406	P	P	P	hsa_circ_0027493	circRNA	ATTAGTGAGACA
hsa_circ_0035768	5.159908059	5.785593314	6.249715853	P	P	P	hsa_circ_0035768	circRNA	ATTAAGCAAGGT
hsa_circ_0082477	10.11508209	10.34651041	10.50329291	P	P	P	hsa_circ_0082477	circRNA	AACGTGTTGCAG
hsa_circ_0006417	9.133661429	10.07444239	9.852210089	P	P	P	hsa_circ_0006417	circRNA	AACCTTCCCTGT
hsa_circ_0010126	5.623513693	5.266317682	5.706331254	P	P	P	hsa_circ_0010126	circRNA	TCACTGAGTACA
hsa_circ_0055559	4.252778069	4.80335802	4.887532663	A	A	A	hsa_circ_0055559	circRNA	AGTCATACAGGT
hsa_circ_0035780	5.517128529	6.114382643	5.834303416	P	P	P	hsa_circ_0035780	circRNA	TTACTATCAGAA
hsa_circ_0034646	10.07650774	9.921101751	9.858868596	P	P	P	hsa_circ_0034646	circRNA	TGTCCCATACTG
hsa_circ_0002677	7.555291483	7.839765007	7.801366921	P	P	P	hsa_circ_0002677	circRNA	TAATAAAAAGGG
hsa_circ_0081285	6.191336488	6.161597704	6.104153411	P	P	P	hsa_circ_0081285	circRNA	AGAGGTGAAGCT
hsa_circ_0023854	8.731386299	8.834718431	8.923043326	P	P	P	hsa_circ_0023854	circRNA	AATTGAAACAGC
hsa_circ_0055279	7.083487837	6.362416292	6.507183717	P	P	P	hsa_circ_0055279	circRNA	TTTAATAAAACA
hsa_circ_0015420	6.840300659	6.844234674	6.926831259	P	P	P	hsa_circ_0015420	circRNA	TTAGCACCAGCT
hsa_circ_0051796	7.004894784	6.921571014	7.361367019	P	P	P	hsa_circ_0051796	circRNA	ACAAACGCTGAT
hsa_circ_0005057	5.912376859	6.0441828	6.363439571	P	P	P	hsa_circ_0005057	circRNA	TTCCATAATCAG
hsa_circ_0016695	5.187758181	5.50024341	5.624175875	P	P	P	hsa_circ_0016695	circRNA	TATTCACATAGT
hsa_circ_0022568	7.310000586	6.751205538	6.580259597	P	P	P	hsa_circ_0022568	circRNA	ATGACAGTCTGC
hsa_circ_0064557	4.891000811	5.192398296	5.464891983	P	P	P	hsa_circ_0064557	circRNA	CCTTTCACAGGT
hsa_circ_0002830	6.243481059	6.077861154	6.223693126	P	P	P	hsa_circ_0002830	circRNA	GATAATTCGAGA
hsa_circ_0017031	6.22062897	5.615834659	5.272078882	P	P	P	hsa_circ_0017031	circRNA	GCCCAAGATGGA
hsa_circ_0017084	6.324588465	6.291597081	6.274187754	P	P	P	hsa_circ_0017084	circRNA	TGTTATTAAAGT
hsa_circ_0085102	6.027773329	6.237121763	6.0665539	P	P	P	hsa_circ_0085102	circRNA	GATTAAATTACT
hsa_circ_0057623	4.758925222	5.444383758	5.482965116	P	P	P	hsa_circ_0057623	circRNA	TCATTCAGAATG
hsa_circ_0035745	5.725246906	5.756382455	5.925390614	P	P	P	hsa_circ_0035745	circRNA	GTGTATGAAGGT
[illegible]	[illegible]	[illegible]	[illegible]	[illegible]	[illegible]	[illegible]	[illegible]	[illegible]	[illegible]

In the rows above, each of the Annotation columns Accession, Source, chr, strand, start, end, GeneSymbol, gene_description, EntrezGeneID, Go, Relation, Associated_gene, Associated_gene_description, Associated_gene_strand reads NA.

Probe ID	circRNA_ID	chrom	hg19_circRNA_start	hg19_circRNA_end	hg19_circRNA_strand	genomic_length	spliced_seq_length	samples	repeats	annotation	best_transcript	gene_symbol	circRNA_study
hsa_circ_0019389	hsa_circ_0019389	chr10	99946223	99969656	+	23433	23433	"""K562, Gm12878	NA	"""ALT_ACCEPTO	NM_014472	C10orf28	Salzman 2013
hsa_circ_0034233	hsa_circ_0034233	chr15	28459800	28501463	-	41663	3841	"""K562, A549"""	NA	"""ANNOTATED	NM_004667	HERC2	Salzman 2013
hsa_circ_0024013	hsa_circ_0024013	chr11	93428662	93463522	+	34860	6360	K562	NA	"""ANNOTATED	NM_033395	KIAA1731	Salzman 2013
hsa_circ_0015764	hsa_circ_0015764	chr1	197091517	197094085	-	2568	516	"""Huvec, Helas3""	NA	"""ANNOTATED	NM_018136	ASPM	Salzman 2013
hsa_circ_0011134	hsa_circ_0011134	chr1	28600550	28609002	+	8452	2231	"""K562, Ag04450	NA	"""ANNOTATED	NM_031459	SESN2	Salzman 2013
hsa_circ_0034042	hsa_circ_0034042	chr15	20663485	20663700	-	215	215	A549	NA	"""ANNOTATED	NR_036432	HERC2P3	Salzman 2013
hsa_circ_0023852	hsa_circ_0023852	chr11	82875248	82875443	+	195	195	"""K562, Gm12878	NA	"""ANNOTATED	NM_015885	PCF11	Salzman 2013
hsa_circ_0035807	hsa_circ_0035807	chr15	64039922	64050568	-	10646	1328	K562	NA	"""ANNOTATED	NM_003922	HERC1	Salzman 2013
hsa_circ_0084230	hsa_circ_0084230	chr8	48685668	48803041	-	117373	9605	"""K562, Bj"""	NA	"""ANNOTATED	NM_006904	PRKDC	Salzman 2013
hsa_circ_0027493	hsa_circ_0027493	chr12	69218142	69218431	+	289	165	"""Nhek, K562, He	NA	"""ANNOTATED	NM_002392	MDM2	Salzman 2013
hsa_circ_0035768	hsa_circ_0035768	chr15	63966540	64050568	-	84028	6820	K562	NA	"""ANNOTATED	NM_003922	HERC1	Salzman 2013
hsa_circ_0082477	hsa_circ_0082477	chr7	134222945	134223769	+	824	167	A549	NA	"""ANNOTATED	NM_020299	AKR1B10	Salzman 2013
hsa_circ_0006417	hsa_circ_0006417	chr11	65202523	65205128	+	2605	2605	"""Hs68_RNase, H	None	INTERGENIC	None	None	Jeck 2013
hsa_circ_0010126	hsa_circ_0010126	chr1	16458872	16460410	-	1538	433	Hepg2	NA	"""ANNOTATED	NM_004431	EPHA2	Salzman 2013
hsa_circ_0055559	hsa_circ_0055559	chr2	88930917	88931337	+	420	420	Helas3	LINE	"""ANNOTATED	TCONS_00002711	TCONS_00002711	Salzman 2013
hsa_circ_0035780	hsa_circ_0035780	chr15	63970043	64021567	-	51524	4049	Huvec	NA	"""ANNOTATED	NM_003922	HERC1	Salzman 2013
hsa_circ_0034646	hsa_circ_0034646	chr15	41247747	41248717	+	970	970	"""Helas3, Ag0445	NA	"""ANNOTATED	NM_001142776	CHAC1	Salzman 2013
hsa_circ_0002677	hsa_circ_0002677	chr17	58733959	58734403	+	444	444	"""Hs68_RNase, H	None	"""ALT_DONOR	NM_003620	PPM1D	Jeck 2013
hsa_circ_0081285	hsa_circ_0081285	chr7	98533201	98569568	+	36367	3804	"""Helas3, H1hesc,	NA	"""ANNOTATED	NM_001244580	TRRAP	Salzman 2013
hsa_circ_0023854	hsa_circ_0023854	chr11	82876641	82888861	+	12220	3281	K562	NA	"""ANNOTATED	NM_015885	PCF11	Salzman 2013
hsa_circ_0055279	hsa_circ_0055279	chr2	74425689	74442424	+	16735	2285	K562	NA	"""ANNOTATED	NR_027405	MTHFD2	Salzman 2013
hsa_circ_0015420	hsa_circ_0015420	chr1	179079416	179084165	-	4749	417	Ag04450	NA	"""ANNOTATED	NM_007314	ABL2	Salzman 2013
hsa_circ_0051796	hsa_circ_0051796	chr19	49438256	49448226	+	9970	951	K562	NA	"""ANNOTATED	NM_014475	DHDH	Salzman 2013
hsa_circ_0005057	hsa_circ_0005057	chr1	33403997	33415375	-	11378	1110	"""Hs68_RNase, H	None	"""ANNOTATED	NM_153341	RNF19B	"""Jeck 2013, Salzman 2013"""
hsa_circ_0016695	hsa_circ_0016695	chr1	226170402	226180703	-	10301	3718	K562	LINE	"""ANNOTATED	NM_152608	C1orf55	Salzman 2013
hsa_circ_0022568	hsa_circ_0022568	chr11	62621990	62623217	-	1227	490	"""K562, Hepg2, H	NA	"""ALT_ACCEPTO	NR_003098	SNHG1	Salzman 2013
hsa_circ_0064557	hsa_circ_0064557	chr3	18456602	18462483	-	5881	663	H1hesc	NA	"""ANNOTATED	NM_001195470	SATB1	Salzman 2013
hsa_circ_0002830	hsa_circ_0002830	chr15	20662557	20663700	-	1143	672	"""Hs68_RNase, H	None	"""ANNOTATED	NR_036432	HERC2P3	"""Jeck 2013, Salzman 2013"""
hsa_circ_0017031	hsa_circ_0017031	chr1	234582548	234593502	-	10954	502	Hepg2	NA	"""ANNOTATED	NM_005646	TARBP1	Salzman 2013
hsa_circ_0017084	hsa_circ_0017084	chr1	235920580	235938386	-	17806	1599	A549	NA	"""ANNOTATED	NM_000081	LYST	Salzman 2013
hsa_circ_0085102	hsa_circ_0085102	chr8	100789002	100889808	+	100806	6661	Gm12878	NA	"""ANNOTATED	NM_017890	VPS13B	Salzman 2013
hsa_circ_0057623	hsa_circ_0057623	chr2	197975489	197987516	-	12027	435	Gm12878	NA	"""ANNOTATED	NM_001195144	ANKRD44	Salzman 2013
hsa_circ_0035745	hsa_circ_0035745	chr15	63953171	64050568	-	97397	8197	"""K562, Gm12878	NA	"""ANNOTATED	NM_003922	HERC1	Salzman 2013
[illegible]	[illegible]	[illegible]	[illegible]	[illegible]	[illegible]	[illegible]	[illegible]	[illegible]	[illegible]	[illegible]	[illegible]	[illegible]	[illegible]
