# Supplementary material for: Oxidative Stress-Induced circHBEGF Promotes Extracellular Matrix Production via Regulating miR-646/EGFR in Human Trabecular Meshwork Cells
Source: Oxid Med Cell Longev. 2020 Nov 24;2020:4692034. doi: 10.1155/2020/4692034 (PMC7722639; doi:10.1155/2020/4692034)
Supplement: Supplementary 4 — Table S2: differentially expressed mRNAs. [file 4692034.f4.pdf]

Column A: Title ;
Column B: Mean ;
Column C: Example；

Title	Mean	Example
Title	Mean	Example
ControlType	quality control site（pos:Quality Control Point，FALSE: Non-Q	Pos/FALSE
Sample_NS	Normalized signal values of probes in samples	7.023509
Sample_flag	Detection and determination of samples：A：There is no signifi	P/M/A
Fold_change(1_vs_2)	（formula : foldchange=average(power(2,signal(CASE)))/ avera	3.4
foldchange (abs)	（formula : if foldchange >1, foldchange (abs) = foldchange，fc	3.4
Regulation(1_vs_2)	Up-down trend of two groups (Biologically repetitive samples) o	up/down
Type	RNAtype：circRNA/lncRNA / mRNA	circRNA/lncRNA/mRNA
ProbeSeq	Probe sequence	AAGGTATCTTAAGGAAAGTTCAAGGTAAAAAGACAGAGGTGTTTATGGTTACATGTGTGT
Accession	ID of RNA in database	lnc-ZNF558-1:4
Source	Database Sources of Gene Sequences	ENSEMBL_GENCODE
Chr	Chromosome Location	Chr19
strand	Sequence Positive and Negative Chain Sources	-/+
Start	Sequence starting position	8831143
end	Sequence termination position	8832242
GeneSymbol	Gene name abbreviation	SNHG23
gene_description	Gene Function Description	translation regulatory long non-coding RNA 1
EntrezGeneID	Number of genes on Entrez	55357
GO	gene ontology: classification of genes according to cellular biolo	GO:0043565
Relation	The relationship between lncRNA and adjacent mRNA	Intergenic
Associated_gene	Gene symbols for identified lncRNA associated genes in relation	Zfp846

Fold-Change cut-off: 2.0
P-value cut-off: 0.05
compare: g2 VS g1
foldchange:the ratio (not logarithm) of normalized intensities between two conditions ; Formula : foldchange=average(power(2,signal(CASE)))/average(power(2,signal(CONTROL))).
foldchange (abs):the absolute ratio (not logarithm) of normalized intensities between two conditions ; Formula : if foldchange>1,foldchange(abs)=foldchange,foldchange<1,foldchange(abs)=1/foldchange.

Probe ID	T test p-value	Fold Change			Normalized signal (log2) of g2			Detection of g2			Normalized signal (log2) of g1			Detection of g1			Annotation																													
ProbeName	pvalues	foldchange	foldchange(abs)	Regulation	1_HTMC_H2O2_N	2_HTMC_H2O2_N	3_HTMC_H2O2_N	HTMC_H2O2_fl	HTMC_H2O2_fl	HTMC_H2O2_fl	S1_HTMC_no_NS	S2_HTMC_no_NS	S3_HTMC_no_NS	1_HTMC_no_flag	2_HTMC_no_flag	3_HTMC_no_flag	ProbeName.1	Type	ProbeSeq	Accession	Source	chr	strand	start	end	GeneSymbol	gene_description	EntrezGeneID	Go	Relation	Associated_gene	iated_gene_descri	sociated_gene_stra	circRNA_ID	chrom	_hg19_circRNA_s	hg19_circRNA_	hg19_circRNA_st	genomic_length	spliced_seq_length	samples	repeats	annotation	best_transcript	gene_symbol	circRNA_study
LNCV6_137244_PI43004817	0.003281265	4.755206315	4.755206315	up	9.678050361	9.629924539	9.663604622	P	P	P	7.163990751	7.387966754	7.633314953	P	P	P	LNCV6_137244_P	mRNA	GTAGTCCTGTG	NM_032505	RefSeq	chr3	+	66998302	67011208	KBTBD8	kelch repeat and B	84541	GO:0005794|GO:0	.	.	-	.	NA	NA	NA	NA	NA	NA	NA	NA	NA	NA	NA	NA	NA
LNCV6_129832_PI43004817	0.024773941	2.689382165	2.689382165	up	5.34730287	5.298247429	5.235487612	P	P	P	3.887405204	3.378750504	4.215384929	A	A	A	LNCV6_129832_P	mRNA	GGGCCATATTT	NM_001297617	RefSeq	chr5	-	60751788	60844274	ELOVL7	ELOVL fatty acid	79993	GO:0005515|GO:0	.	.	-	.	NA	NA	NA	NA	NA	NA	NA	NA	NA	NA	NA	NA	NA
LNCV6_65532_PI430048170	0.004550273	2.003714043	2.003714043	up	6.27291688	6.402542642	6.251232653	P	P	P	5.086352034	5.419609227	5.394145338	P	A	A	LNCV6_65532_PI	mRNA	GAGCAATTTTT	NM_199346	RefSeq	chr2	-	24114808	24123477	PFN4	profilin family%2C	375189	GO:0005737|GO:0	.	.	-	.	NA	NA	NA	NA	NA	NA	NA	NA	NA	NA	NA	NA	NA
LNCV6_132256_PI43004817	0.000434836	2.263539897	2.263539897	up	8.134861716	7.998002608	8.012713547	P	P	P	6.845319155	6.898702148	6.869267587	P	P	P	LNCV6_132256_P	mRNA	GTGTCTTCACA	NM_177987	RefSeq	chr10	-	46887	49238	TUBB8	tubulin%2C beta 8	347688	GO:0006184|GO:0	.	.	-	.	NA	NA	NA	NA	NA	NA	NA	NA	NA	NA	NA	NA	NA
LNCV6_142859_PI43004817	0.000931441	3.997934078	3.997934078	up	6.421002698	6.466270698	6.668659393	P	P	P	4.498590648	4.757728771	4.273282384	A	A	A	LNCV6_142859_P	mRNA	CGCGGTATATA	NM_023004	RefSeq	chr22	-	20241414	20268293	RTN4R	reticulon 4 receptor	65078	GO:0005515|GO:0	.	.	-	.	NA	NA	NA	NA	NA	NA	NA	NA	NA	NA	NA	NA	NA
LNCV6_129100_PI43004817	0.02947684	2.195257004	2.195257004	up	6.253580079	6.213504537	5.992284242	P	P	P	5.446298198	4.640014183	4.859140075	P	A	A	LNCV6_129100_P	mRNA	TACCTGCCTGG	NM_016335	RefSeq	chr22_KI270734v1	-	138078	161852	PRODH	proline dehydroger	5625	GO:0071949|GO:0	.	.	-	.	NA	NA	NA	NA	NA	NA	NA	NA	NA	NA	NA	NA	NA
LNCV6_143175_PI43004817	0.022270082	3.81643899	3.81643899	up	5.104846965	5.151357501	5.317165601	P	P	P	2.844839596	2.864220488	3.838934628	A	A	A	LNCV6_143175_P	mRNA	ATGGATCTCAT	NM_001168214	RefSeq	chr3	+	160225635	160228213	C3orf80	chromosome 3 ope	401097	GO:0016021	.	.	-	.	NA	NA	NA	NA	NA	NA	NA	NA	NA	NA	NA	NA	NA
LNCV6_133942_PI43004817	0.001265016	0.39544055	2.528825127	down	6.787953085	7.178227454	6.866422796	P	P	P	8.055282616	8.395228784	8.401237505	P	P	P	LNCV6_133942_P	mRNA	GCTCTTTGAGG	NM_018651	RefSeq	chr3	+	44555174	44572158	ZKSCAN7	zinc finger with KR	55888	GO:0006355|GO:0	.	.	-	.	NA	NA	NA	NA	NA	NA	NA	NA	NA	NA	NA	NA	NA
LNCV6_127988_PI43004817	3.57406E-05	0.375894747	2.660319163	down	9.237928141	9.285348541	9.371865417	P	P	P	10.77214244	10.74430338	10.61166006	P	P	P	LNCV6_127988_P	mRNA	TGGGGATGAAT	NM_001809	RefSeq	chr2	+	26786013	26794587	CENPA	centromere protein	1058	GO:0005515|GO:0	.	.	-	.	NA	NA	NA	NA	NA	NA	NA	NA	NA	NA	NA	NA	NA
LNCV6_136272_PI43004817	0.000280786	2.435237076	2.435237076	up	11.90306232	11.85734359	11.66391718	P	P	P	10.42892105	10.54963612	10.59930105	P	P	P	LNCV6_136272_P	mRNA	AGCCACTGGGG	NM_004556	RefSeq	chr6	-	44258165	44265788	NFKBIE	nuclear factor of ka	4794	GO:0005515|GO:0	.	.	-	.	NA	NA	NA	NA	NA	NA	NA	NA	NA	NA	NA	NA	NA
LNCV6_138439_PI43004817	0.004396472	2.054842174	2.054842174	up	8.09431288	7.900836022	8.118874818	P	P	P	7.201764861	6.773849113	6.999438592	P	P	P	LNCV6_138439_P	mRNA	AAATGGCATAG	NM_014143	RefSeq	chr9	+	5450502	5470567	CD274	CD274 molecule%	29126	GO:0005515|GO:2	.	.	-	.	NA	NA	NA	NA	NA	NA	NA	NA	NA	NA	NA	NA	NA
LNCV6_144437_PI43004817	0.010854164	2.579888922	2.579888922	up	6.031659303	5.574776112	5.557718967	P	P	P	4.318888744	4.419792464	4.373505883	A	A	A	LNCV6_144437_P	mRNA	GCTGAGCCGGC	NM_173576	RefSeq	chr10	-	27672873	27745849	MKX	mohawk homeobox	283078	GO:0006366|GO:0	.	.	-	.	NA	NA	NA	NA	NA	NA	NA	NA	NA	NA	NA	NA	NA
LNCV6_127950_PI43004817	0.000839632	6.377652442	6.377652442	up	5.191758187	4.7973289	5.472809526	P	P	P	2.734748128	2.416962528	2.338335982	A	A	A	LNCV6_127950_P	mRNA	GCCCATGTTTC	NM_032562	RefSeq	chr10	-	72935179	72954752	PLA2G12B	phospholipase A2%	84647	GO:0042632|GO:0	.	.	-	.	NA	NA	NA	NA	NA	NA	NA	NA	NA	NA	NA	NA	NA
LNCV6_49460_PI430048170	0.001558947	2.403431729	2.403431729	up	6.720041873	6.429438238	6.772245239	P	P	P	5.129592064	5.477023985	5.512754893	P	P	P	LNCV6_49460_PI	mRNA	TGGAAGATCAG	NM_001195243	RefSeq	chr7	+	129502478	129512932	SMKR1	small lysine-rich pr	100287482	NA	.	.	-	.	NA	NA	NA	NA	NA	NA	NA	NA	NA	NA	NA	NA	NA
LNCV6_140186_PI43004817	0.007084027	3.008886213	3.008886213	up	10.68752659	10.83941377	10.6466985	P	P	P	8.784797426	9.392893563	9.171279981	P	P	P	LNCV6_140186_P	mRNA	GCTGCACAGTC	NM_001039792	RefSeq	chr9	+	35906191	35907141	HRCT1	histidine rich carbo	646962	GO:0016021	.	.	-	.	NA	NA	NA	NA	NA	NA	NA	NA	NA	NA	NA	NA	NA
LNCV6_131376_PI43004817	0.003993093	2.625441062	2.625441062	up	9.416605648	8.924576092	9.095588095	P	P	P	7.893076588	7.664761568	7.735558844	P	P	P	LNCV6_131376_P	mRNA	GACAAACGTGT	NM_052957	RefSeq	chrX	+	71578023	71613583	ACRC	acidic repeat contai	93953	GO:0005634	.	.	-	.	NA	NA	NA	NA	NA	NA	NA	NA	NA	NA	NA	NA	NA
LNCV6_128407_PI43004817	4.8118E-06	18.89008024	18.89008024	up	9.621941294	9.600754602	9.873719629	P	P	P	5.323672821	5.512884389	5.547558564	P	P	P	LNCV6_128407_P	mRNA	GCCCTCCTGAA	NM_002507	RefSeq	chr17	+	49495292	49515020	NGFR	nerve growth factor	4804	GO:0005515|GO:0	.	.	-	.	NA	NA	NA	NA	NA	NA	NA	NA	NA	NA	NA	NA	NA
LNCV6_130421_PI43004817	1.30224E-05	5.581794623	5.581794623	up	8.629809091	8.544811786	8.399854389	P	P	P	5.99342023	6.152931924	5.989124698	P	P	P	LNCV6_130421_P	mRNA	CCTCTCTGTAT	NM_003862	RefSeq	chr5	+	171419662	171457626	FGF18	fibroblast growth f	8817	GO:0008286|GO:0	.	.	-	.	NA	NA	NA	NA	NA	NA	NA	NA	NA	NA	NA	NA	NA
LNCV6_130206_PI43004817	0.000191466	16.57901287	16.57901287	up	8.700806068	8.456401648	8.710714957	P	P	P	4.594474502	4.264349764	4.816625048	P	A	A	LNCV6_130206_P	mRNA	TAGAGAACATG	NM_001737	RefSeq	chr5	-	39284275	39364553	C9	complement compo	735	GO:0006957|GO:0	.	.	-	.	NA	NA	NA	NA	NA	NA	NA	NA	NA	NA	NA	NA	NA
LNCV6_141099_PI43004817	9.54617E-05	2.695544813	2.695544813	up	11.17358043	11.34194203	11.27124474	P	P	P	9.708101371	9.924737547	9.858671966	P	P	P	LNCV6_141099_P	mRNA	GTTTTAAACTC	NM_004295	RefSeq	chr17	+	28744004	28750958	TRAF4	TNF receptor-assoc	9618	GO:0005515|GO:0	.	.	-	.	NA	NA	NA	NA	NA	NA	NA	NA	NA	NA	NA	NA	NA
LNCV6_67040_PI430048170	0.003100196	3.326509365	3.326509365	up	5.539763479	5.937140537	6.213655069	P	P	P	3.978772074	3.971601797	4.539753038	A	A	A	LNCV6_67040_PI	mRNA	CTGTTGGAGTC	NM_194298	RefSeq	chr10	-	59650763	59709891	SLC16A9	solute carrier famil	220963	GO:0015718|GO:0	.	.	-	.	NA	NA	NA	NA	NA	NA	NA	NA	NA	NA	NA	NA	NA
LNCV6_141862_PI43004817	0.028344316	0.408037813	2.450753258	down	4.352469114	4.328911722	4.294879614	P	A	A	5.153022718	5.748458684	5.859987391	P	P	P	LNCV6_141862_P	mRNA	GTTGTAGTTTG	NM_001172655	RefSeq	chr19	+	52570663	52587174	ZNF701	zinc finger protein	55762	GO:0006355|GO:0	.	.	-	.	NA	NA	NA	NA	NA	NA	NA	NA	NA	NA	NA	NA	NA
LNCV6_128833_PI43004817	0.02847963	3.179823868	3.179823868	up	4.605421537	5.326425652	4.728164875	P	P	P	3.760363789	2.415286298	3.28148842	A	A	A	LNCV6_128833_P	mRNA	CATGACACTGA	NM_152538	RefSeq	chr3	-	118900630	119146051	IGSF11	immunoglobulin su	152404	GO:0005886|GO:0	.	.	-	.	NA	NA	NA	NA	NA	NA	NA	NA	NA	NA	NA	NA	NA
LNCV6_135871_PI43004817	1.51264E-05	16.23674397	16.23674397	up	9.949434207	9.681276778	9.751300298	P	P	P	5.739471002	5.868119439	5.719888732	P	P	P	LNCV6_135871_P	mRNA	TACCCCAGAGA	NM_015193	RefSeq	chr8	-	142611043	142614472	ARC	activity-regulated c	23237	GO:0030054|GO:0	.	.	-	.	NA	NA	NA	NA	NA	NA	NA	NA	NA	NA	NA	NA	NA
LNCV6_137787_PI43004817	0.002509381	3.168649698	3.168649698	up	12.0735391	11.69814792	11.89423092	P	P	P	10.29171597	10.2061379	10.19895532	P	P	P	LNCV6_137787_P	mRNA	GCTTGAGATAT	NM_015885	RefSeq	chr11	+	83157094	83185793	PCF11	PCF11 cleavage an	51585	GO:0008380|GO:0	.	.	-	.	NA	NA	NA	NA	NA	NA	NA	NA	NA	NA	NA	NA	NA
LNCV6_2013_PI430048170	0.00505406	0.484680019	2.063216886	down	9.119762604	9.324204779	9.282362848	P	P	P	10.05282881	10.47514333	10.3100157	P	P	P	LNCV6_2013_PI4	mRNA	CAATTACGCTG	NM_001135599	RefSeq	chr1	+	218345333	218444619	TGFB2	transforming growt	7042	GO:0005515|GO:0	.	.	-	.	NA	NA	NA	NA	NA	NA	NA	NA	NA	NA	NA	NA	NA
LNCV6_137050_PI43004817	0.000410882	3.051202991	3.051202991	up	6.167378001	6.188721431	5.910960503	P	P	P	4.467778446	4.403764841	4.578172694	P	A	A	LNCV6_137050_P	mRNA	CCACAGGGAAG	NM_000196	RefSeq	chr16	+	67431132	67437551	HSD11B2	hydroxysteroid (11	3291	GO:0001666|GO:0	.	.	-	.	NA	NA	NA	NA	NA	NA	NA	NA	NA	NA	NA	NA	NA
[illegible]																																														

LNCV6_135894_PI43004817	0.000128434	2.206719868	2.206719868	up	12.03989508	12.0937091	11.97486566	P	P	P	10.83982643	10.844286	10.99558604	P	P	P	LNCV6_135894_F	mRNA	GGAGCAACTTT	NM_004831	RefSeq	chr19	-	16574906	16628204	MED26	mediator complex s	9441	GO:0005515|GO:0	.	.	-	.	NA	NA	NA	NA	NA	NA	NA	NA	NA	NA	NA	NA	NA
LNCV6_135686_PI43004817	0.020871458	2.514605168	2.514605168	up	6.316262371	6.168243014	6.139803546	P	P	P	4.606259708	4.633534671	5.290985421	P	A	P	LNCV6_135686_F	mRNA	GGTTTTCTTTCA	NM_006794	RefSeq	chr2	-	53852912	53860033	GPR75	G protein-coupled r	10936	GO:0070098|GO:0	.	.	-	.	NA	NA	NA	NA	NA	NA	NA	NA	NA	NA	NA	NA	NA
LNCV6_134724_PI43004817	0.000217735	2.063408057	2.063408057	up	11.32652564	11.32478192	11.14598488	P	P	P	10.11447364	10.26402322	10.28513323	P	P	P	LNCV6_134724_F	mRNA	GAAACTATTTA	NM_198850	RefSeq	chr19	-	43475102	43504833	PHLDB3	pleckstrin homolog	653583	GO:0019899	.	.	-	.	NA	NA	NA	NA	NA	NA	NA	NA	NA	NA	NA	NA	NA
LNCV6_132155_PI43004817	0.00409713	4.763565385	4.763565385	up	5.888888972	6.282173723	5.879766612	P	P	P	3.517177284	3.435596638	4.237956638	A	A	A	LNCV6_132155_F	mRNA	GAGTTCACAGT	NM_144715	RefSeq	chr3	-	19879473	19934214	EFHB	EF-hand domain fa	151651	GO:0005509	.	.	-	.	NA	NA	NA	NA	NA	NA	NA	NA	NA	NA	NA	NA	NA
LNCV6_142587_PI43004817	0.011466354	0.41291529	2.421804237	down	5.3950504	5.157307048	5.801461342	P	P	P	6.319799512	7.045394369	6.799980056	P	P	P	LNCV6_142587_F	mRNA	GTACTATGTGC	NM_018557	RefSeq	chr2	-	140231426	142131701	LRP1B	low density lipopro	53353	GO:0043235|GO:0	.	.	-	.	NA	NA	NA	NA	NA	NA	NA	NA	NA	NA	NA	NA	NA
LNCV6_136213_PI43004817	0.00060667	2.143324582	2.143324582	up	6.343882187	6.096384961	6.224993399	P	P	P	5.040865552	5.034942061	5.286015234	P	P	P	LNCV6_136213_F	mRNA	GCTTCTCAAGC	NM_001169	RefSeq	chr16	+	25216963	25228932	AQP8	aquaporin 8	343	GO:0005886|GO:0	.	.	-	.	NA	NA	NA	NA	NA	NA	NA	NA	NA	NA	NA	NA	NA
LNCV6_132957_PI43004817	1.85208E-05	4.208629068	4.208629068	up	10.05618257	9.98814554	10.03844931	P	P	P	7.967355293	7.952595175	7.943536053	P	P	P	LNCV6_132957_F	mRNA	AATCTGACAAA	NM_003885	RefSeq	chr17	+	32487086	32491253	CDK5R1	cyclin-dependent k	8851	GO:0005515|GO:0	.	.	-	.	NA	NA	NA	NA	NA	NA	NA	NA	NA	NA	NA	NA	NA
LNCV6_137143_PI43004817	0.002160473	2.542356439	2.542356439	up	7.740289316	7.561433194	7.670773194	P	P	P	6.157306105	6.230354567	6.525126171	P	P	P	LNCV6_137143_F	mRNA	AAAGAATCTCC	NM_001144875	RefSeq	chr5	-	177501904	177510426	DOK3	docking protein 3%	79930	GO:0005515|GO:0	.	.	-	.	NA	NA	NA	NA	NA	NA	NA	NA	NA	NA	NA	NA	NA
LNCV6_142740_PI43004817	0.005349197	2.246253563	2.246253563	up	7.941391973	7.964336728	8.051801036	P	P	P	6.588733697	6.933627124	6.909934075	P	P	P	LNCV6_142740_F	mRNA	CACATTTTTAA	NM_001142393	RefSeq	chr6	-	11183297	11382348	NEDD9	neural precursor ce	4739	GO:0005515|GO:0	.	.	-	.	NA	NA	NA	NA	NA	NA	NA	NA	NA	NA	NA	NA	NA
LNCV6_129461_PI43004817	0.005801671	2.187471363	2.187471363	up	6.635447268	6.610118357	6.409708605	P	P	P	5.57699394	5.529557573	5.131197233	P	P	P	LNCV6_129461_F	mRNA	CTCTGCTTTTCC	NM_001956	RefSeq	chr1	-	41478774	41484683	EDN2	endothelin 2%2C tr	1907	GO:0008284|GO:0	.	.	-	.	NA	NA	NA	NA	NA	NA	NA	NA	NA	NA	NA	NA	NA
LNCV6_75488_PI430048170	0.000998551	2.315472957	2.315472957	up	9.857939564	9.95557116	9.615442874	P	P	P	8.404614081	8.705415016	8.686681962	P	P	P	LNCV6_75488_PI	mRNA	AGGCTAGAACC	NM_006622	RefSeq	chr5	-	58453982	58460139	PLK2	polo-like kinase 2%	10769	GO:0005515|GO:0	.	.	-	.	NA	NA	NA	NA	NA	NA	NA	NA	NA	NA	NA	NA	NA
LNCV6_137590_PI43004817	2.85235E-05	2.41241621	2.41241621	up	14.78537422	14.92802296	14.87587667	P	P	P	13.51547317	13.60022615	13.66199293	P	P	P	LNCV6_137590_F	mRNA	GTTGGGGGAGC	NM_016545	RefSeq	chr1	+	181088501	181090843	IER5	immediate early res	51278	NA	.	.	-	.	NA	NA	NA	NA	NA	NA	NA	NA	NA	NA	NA	NA	NA
LNCV6_142884_PI43004817	0.012965272	2.984861732	2.984861732	up	7.465307635	7.447489383	7.353257266	P	P	P	5.439809446	5.905382753	6.111044984	P	P	P	LNCV6_142884_F	mRNA	AGCTACCTTTT	NM_001271562	RefSeq	chr11	-	114398533	114400550	C11orf71	chromosome 11 op	54494	NA	.	.	-	.	NA	NA	NA	NA	NA	NA	NA	NA	NA	NA	NA	NA	NA
LNCV6_136693_PI43004817	0.000945343	3.467962581	3.467962581	up	15.32785879	15.34365071	15.34018711	P	P	P	13.52093476	13.64852555	13.45315564	P	P	P	LNCV6_136693_F	mRNA	TCTTTGGCTTTC	NM_031459	RefSeq	chr1	+	28259451	28282491	SESN2	sestrin 2	83667	GO:0005515|GO:0	.	.	-	.	NA	NA	NA	NA	NA	NA	NA	NA	NA	NA	NA	NA	NA
LNCV6_63460_PI430048170	0.002978328	2.300545343	2.300545343	up	6.186160603	6.638307942	6.374568885	P	P	P	5.208871101	5.060358291	5.345772708	P	P	P	LNCV6_63460_PI	mRNA	TGTCAGTATTCT	NM_001162995	RefSeq	chr17	+	75633433	75641406	SMIM5	small integral mem	NA	NA	.	.	-	.	NA	NA	NA	NA	NA	NA	NA	NA	NA	NA	NA	NA	NA
LNCV6_138007_PI43004817	0.001184182	2.389662196	2.389662196	up	10.88746016	10.95413511	10.8711891	P	P	P	9.774768669	9.534097862	9.624536893	P	P	P	LNCV6_138007_F	mRNA	CTCCGCAGCCT	NM_024640	RefSeq	chr1	-	37802941	37808193	YRDC	yrdC N(6)-threonyl	79693	GO:0005739|GO:0	.	.	-	.	NA	NA	NA	NA	NA	NA	NA	NA	NA	NA	NA	NA	NA
LNCV6_132792_PI43004817	0.00243851	2.137817274	2.137817274	up	6.830661481	7.107289623	6.748956113	P	P	P	5.972872405	5.573704137	5.848411204	P	P	P	LNCV6_132792_F	mRNA	GAACCCTGCAA	NM_001146288	RefSeq	chr22	+	20967158	20981360	AIFM3	apoptosis-inducing	150209	GO:0005739|GO:0	.	.	-	.	NA	NA	NA	NA	NA	NA	NA	NA	NA	NA	NA	NA	NA
LNCV6_143301_PI43004817	0.000573505	2.059279664	2.059279664	up	9.443760481	9.403825663	9.524404719	P	P	P	8.453343693	8.289016294	8.497572317	P	P	P	LNCV6_143301_F	mRNA	GCCTTCAATGA	NM_003463	RefSeq	chr6	+	63572011	63583588	PTP4A1	protein tyrosine ph	7803	GO:0007049|GO:0	.	.	-	.	NA	NA	NA	NA	NA	NA	NA	NA	NA	NA	NA	NA	NA
LNCV6_110681_PI43004817	0.003670191	2.682753619	2.682753619	up	7.012923382	6.998941824	6.996006591	P	P	P	5.492860547	5.478281885	5.749235239	P	P	P	LNCV6_110681_F	mRNA	AGACCATAAAT	NM_004490	RefSeq	chr2	-	164492812	164621850	GRB14	growth factor recep	2888	GO:0007165|GO:0	.	.	-	.	NA	NA	NA	NA	NA	NA	NA	NA	NA	NA	NA	NA	NA
LNCV6_136370_PI43004817	0.000209149	2.678441965	2.678441965	up	7.06238673	6.848791193	7.067485094	P	P	P	5.411665602	5.625120201	5.674975149	P	P	P	LNCV6_136370_F	mRNA	CAACGCCTTGT	NM_001010971	RefSeq	chr1	+	84298365	84350798	SAMD13	sterile alpha motif	148418	NA	.	.	-	.	NA	NA	NA	NA	NA	NA	NA	NA	NA	NA	NA	NA	NA
LNCV6_142975_PI43004817	0.000415888	0.440593905	2.269663715	down	9.392721442	9.148574623	9.232989578	P	P	P	10.28129139	10.53990485	10.49813632	P	P	P	LNCV6_142975_F	mRNA	GCGTTGTTACC	NM_025225	RefSeq	chr22	+	43923738	43947568	PNPLA3	patatin-like phosph	80339	GO:0051265|GO:0	.	.	-	.	NA	NA	NA	NA	NA	NA	NA	NA	NA	NA	NA	NA	NA
LNCV6_136717_PI43004817	0.024884518	6.621212233	6.621212233	up	6.301087457	6.237715011	6.258192573	P	P	P	2.533761549	4.109527443	3.562403895	A	A	A	LNCV6_136717_F	mRNA	GACTAGTATAG	NM_033035	RefSeq	chr5	+	111071691	111078024	TSLP	thymic stromal lym	85480	GO:0005615|GO:0	.	.	-	.	NA	NA	NA	NA	NA	NA	NA	NA	NA	NA	NA	NA	NA
LNCV6_127948_PI43004817	0.00423676	2.230232375	2.230232375	up	8.411563031	8.233396066	8.507141514	P	P	P	7.489952564	7.038321674	7.12465899	P	P	P	LNCV6_127948_F	mRNA	CATCTTTCAGA	NM_057158	RefSeq	chr8	-	29333061	29348805	DUSP4	dual specificity pho	1846	GO:0034142|GO:0	.	.	-	.	NA	NA	NA	NA	NA	NA	NA	NA	NA	NA	NA	NA	NA
LNCV6_144012_PI43004817	0.002914065	2.910399644	2.910399644	up	8.778555046	8.747281763	8.867765631	P	P	P	7.02016695	7.33940733	7.38631275	P	P	P	LNCV6_144012_F	mRNA	CTTGTAAAAGC	NM_001291832	RefSeq	chr2	+	238159007	238168891	FAM132B	family with sequen	151176	GO:0006879|GO:0	.	.	-	.	NA	NA	NA	NA	NA	NA	NA	NA	NA	NA	NA	NA	NA
LNCV6_143503_PI43004817	0.037652759	5.25957443	5.25957443	up	6.882934228	6.751824136	6.664203111	P	P	P	3.199088396	4.964953823	4.446085699	A	P	A	LNCV6_143503_F	mRNA	CTCCCGTTAAG	NM_001114938	RefSeq	chr1	-	45620043	45624059	CCDC17	coiled-coil domain	149483	NA	.	.	-	.	NA	NA	NA	NA	NA	NA	NA	NA	NA	NA	NA	NA	NA
LNCV6_142319_PI43004817	0.016475682	4.137762302	4.137762302	up	6.299833997	6.170572032	6.238999466	P	P	P	3.570281996	4.385171237	4.454712526	A	A	A	LNCV6_142319_F	mRNA	AAGCCCTGTGG	NM_001100	RefSeq	chr1	-	229431245	229434096	ACTA1	actin%2C alpha 1%	58	GO:0030240|GO:0	.	.	-	.	NA	NA	NA	NA	NA	NA	NA	NA	NA	NA	NA	NA	NA
LNCV6_143937_PI43004817	0.002387135	3.003053575	3.003053575	up	7.989322304	8.04793821	7.959215091	P	P	P	6.238911727	6.394959114	6.583979555	P	P	P	LNCV6_143937_F	mRNA	CAAAAATTATG	NM_001301838	RefSeq	chr12	+	6943816	6946003	C12orf57	chromosome 12 op	113246	GO:0005737	.	.	-	.	NA	NA	NA	NA	NA	NA	NA	NA	NA	NA	NA	NA	NA
LNCV6_134045_PI43004817	0.030253064	2.01289825	2.01289825	up	6.122386035	6.277545892	6.443897819	P	P	P	5.691902422	4.984481577	5.046515434	P	P	P	LNCV6_134045_F	mRNA	CATGCTTTGTT	NM_033058	RefSeq	chr8	-	66127042	66175483	TRIM55	tripartite motif con	84675	GO:0007165|GO:0	.	.	-	.	NA	NA	NA	NA	NA	NA	NA	NA	NA	NA	NA	NA	NA
LNCV6_2531_PI430048170	0.000157936	4.572721208	4.572721208	up	10.17594947	10.02185073	10.13597871	P	P	P	8.083500513	7.809942795	7.850194028	P	P	P	LNCV6_2531_PI4	mRNA	TGTGTTGTGTG	NM_001286401	RefSeq	chr6	-	37212177	37258155	TMEM217	transmembrane pro	221468	GO:0016021	.	.	-	.	NA	NA	NA	NA	NA	NA	NA	NA	NA	NA	NA	NA	NA
LNCV6_99479_PI430048170	0.001058436	2.741101665	2.741101665	up	11.0205816	10.980467	11.11620673	P	P	P	9.676102118	9.398551676	9.664982662	P	P	P	LNCV6_99479_PI	mRNA	TGACTATGGCT	NM_000963	RefSeq	chr1	-	186671811	186680427	PTGS2	prostaglandin-endo	5743	GO:0019369|GO:0	.	.	-	.	NA	NA	NA	NA	NA	NA	NA	NA	NA	NA	NA	NA	NA
LNCV6_145612_PI43004817	0.007683015	9.630649161	9.630649161	up	7.298955639	7.490742729	7.50657629	P	P	P	4.313472858	4.529668966	3.450020609	A	A	A	LNCV6_145612_F	mRNA	TACCCAGTCCT	NM_012125	RefSeq	chr15	+	33968887	34065086	CHRM5	cholinergic recepto	1133	GO:0030054|GO:0	.	.	-	.	NA	NA	NA	NA	NA	NA	NA	NA	NA	NA	NA	NA	NA
LNCV6_54163_PI430048170	0.012463586	3.603309478	3.603309478	up	6.577298149	6.108108733	6.612588407	P	P	P	3.975040253	4.573104945	5.05626682	A	A	P	LNCV6_54163_PI	mRNA	CTGGCATTCAA	NM_198715	RefSeq	chr1	-	71005853	71047808	PTGER3	prostaglandin E rec	5733	GO:0008219|GO:0	.	.	-	.	NA	NA	NA	NA	NA	NA	NA	NA	NA	NA	NA	NA	NA
LNCV6_136912_PI43004817	0.000579179	4.236334616	4.236334616	up	6.85918711	6.426934306	6.903576207	P	P	P	4.441572007	4.923171608	4.579338467	A	P	A	LNCV6_136912_F	mRNA	AGTGGGAAAGA	NM_145259	RefSeq	chr2	-	157526766	157628887	ACVR1C	activin A receptor	130399	GO:0016361|GO:0	.	.	-	.	NA	NA	NA	NA	NA	NA	NA	NA	NA	NA	NA	NA	NA
LNCV6_132008_PI43004817	0.004724426	2.90333466	2.90333466	up	7.480245058	7.480422262	7.40737853	P	P	P	6.00924463	5.666824654	6.050222077	P	P	P	LNCV6_132008_F	mRNA	AGCCCCTTAAG	NM_019850	RefSeq	chr2	-	232878685	233013241	NGEF	neuronal guanine n	25791	GO:0051056|GO:0	.	.	-	.	NA	NA	NA	NA	NA	NA	NA	NA	NA	NA	NA	NA	NA
LNCV6_140163_PI43004817	0.00070671	0.487473223	2.051394727	down	5.782558619	5.600133022	5.739762836	P	P	P	6.595029641	6.860217676	6.770778621	P	P	P	LNCV6_140163_F	mRNA	TTTCTATAGAA	NM_001297619	RefSeq	chr19	-	36334452	36379203	ZFP14	ZFP14 zinc finger p	57677	GO:0006355|GO:0	.	.	-	.	NA	NA	NA	NA	NA	NA	NA	NA	NA	NA	NA	NA	NA
LNCV6_141847_PI43004817	0.041252718	4.378183846	4.378183846	up	6.121400932	5.820994004	5.80547248	P	P	P	3.076874301	4.62461807	3.084194663	A	A	A	LNCV6_141847_F	mRNA	AACTCATGGGA	NM_015370	RefSeq	chr22	-	29058671	29061919	C22orf31	chromosome 22 op	25770	NA	.	.	-	.	NA	NA	NA	NA	NA	NA	NA	NA	NA	NA	NA	NA	NA
LNCV6_134198_PI43004817	0.000758005	2.170950852	2.170950852	up	7.8580153	7.933567917	8.094514696	P	P	P	6.688518468	6.996170277	6.8402346	P	P	P	LNCV6_134198_F	mRNA	GGAGAAACACC	NM_001194958	RefSeq	chr17	+	21692522	21704612	KCNJ18	potassium channel	NA	NA	.	.	-	.	NA	NA	NA	NA	NA	NA	NA	NA	NA	NA	NA	NA	NA
LNCV6_142039_PI43004817	0.007255103	3.705451797	3.705451797	up	6.766111864	6.693417539	6.722822897	P	P	P	4.974332749	4.476624061	5.004973449	P	A	P	LNCV6_142039_F	mRNA	AAGAGCCTTTA	NM_001199280	RefSeq	chr16	+	69105563	69117667	HAS3	hyaluronan synthas	3038	GO:0005886|GO:0	.	.	-	.	NA	NA	NA	NA	NA	NA	NA	NA	NA	NA	NA	NA	NA
LNCV6_137546_PI43004817	9.4655E-05	7.683712271	7.683712271	up	5.567556754	5.332020909	5.097834726	P	P	P	2.64141736	2.287057089	2.24831046	A	A	A	LNCV6_137546_F	mRNA	CCATGAGACCA	NM_000575	RefSeq	chr2	-	112773914	112785394	IL1A	interleukin 1%2C a	3552	GO:0005515|GO:0	.	.	-	.	NA	NA	NA	NA	NA	NA	NA	NA	NA	NA	NA	NA	NA
LNCV6_144611_PI43004817	0.001365808	2.413606682	2.413606682	up	10.0423771	10.26244392	10.25654838	P	P	P	8.688234745	9.005565979	9.039381665	P	P	P	LNCV6_144611_F	mRNA	CTTTCGGTATG	NM_178012	RefSeq	chr6	-	3224260	3227734	TUBB2B	tubulin%2C beta 2	347733	GO:0005515|GO:0	.	.	-	.	NA	NA	NA	NA	NA	NA	NA	NA	NA	NA	NA	NA	NA
LNCV6_143876_PI43004817	0.000869724	0.420727371	2.376836093	down	10.52188404	10.75738973	10.51081278	P	P	P	11.77561056	11.91552905	11.85634641	P	P	P	LNCV6_143876_F	mRNA	TCTGTGACTCC	NM_001032291	RefSeq	chr1	-	109279553	109283168	PSRC1	proline/serine-rich	84722	GO:0005515|GO:0	.	.	-	.	NA	NA	NA	NA	NA	NA	NA	NA	NA	NA	NA	NA	NA
LNCV6_142961_PI43004817	0.000199954	0.434097565	2.303629599	down	11.85846822	12.04926699	12.02786869	P	P	P	13.10450424	13.31626588	13.12441384	P	P	P	LNCV6_142961_F	mRNA	TGAATACAAGT	NM_001466	RefSeq	chr17	+	44557443	44561262	FZD2	frizzled class recep	2535	GO:0017147|GO:0	.	.	-	.	NA	NA	NA	NA	NA	NA	NA	NA	NA	NA	NA	NA	NA
LNCV6_137889_PI43004817	0.002190101	7.093412716	7.093412716	up	6.08432585	6.201301245	6.289362525	P	P	P	3.132555238	3.1763819	3.717547441	A	A	A	LNCV6_137889_F	mRNA	TTTGAACAACC	NM_000260	RefSeq	chr11	+	77128263	77215241	MYO7A	myosin VIIA%2C t	4647	GO:0005515|GO:0	.	.	-	.	NA	NA	NA	NA	NA	NA	NA	NA	NA	NA	NA	NA	NA
LNCV6_135127_PI43004817	2.06229E-07	22.46161112	22.46161112	up	6.820176686	6.779683246	6.67829219	P	P	P	2.213617285	2.334392028	2.26309101	A	A	A	LNCV6_135127_F	mRNA	CTCCAGTCTGA	NM_182847	RefSeq	chr2	+	219514169	219538772	ASIC4	acid sensing (proto	55515	GO:0034220|GO:0	.	.	-	.	NA	NA	NA	NA	NA	NA	NA	NA	NA	NA	NA	NA	NA
LNCV6_136328_PI43004817	0.005001714	2.148986883	2.148986883	up	13.36267688	12.88544872	12.98289295	P	P	P	12.18558283	11.8810031	11.87640094	P	P	P	LNCV6_136328_F	mRNA	TGCATTTCTTTT	NM_175617	RefSeq	chr16	+	56625672	56627113	MT1E	metallothionein 1E	4493	GO:0071294|GO:0	.	.	-	.	NA	NA	NA	NA	NA	NA	NA	NA	NA	NA	NA	NA	NA
LNCV6_129051_PI43004817	0.001734416	2.887623694	2.887623694	up	9.352077166	9.051398342	8.985904253	P	P	P	7.689538425	7.518205166	7.613880179	P	P	P	LNCV6_129051_F	mRNA	CATTGCTCTTT	NM_173200	RefSeq	chr9	+	99821854	99866891	NR4A3	nuclear receptor su	8013	GO:0005515|GO:0	.	.	-	.	NA	NA	NA	NA	NA	NA	NA	NA	NA	NA	NA	NA	NA
LNCV6_129738_PI43004817	0.03442134	2.018150649	2.018150649	up	5.81650913	5.875005766	5.859086638	P	P	P	4.523072925	4.706443708	5.196359965	P	A	P	LNCV6_129738_F	mRNA	CAACCTTCAGG	NM_005630	RefSeq	chr3	-	133932695	134030076	SLCO2A1	solute carrier organ	6578	GO:0015132|GO:0	.	.	-	.	NA	NA	NA	NA	NA	NA	NA	NA	NA	NA	NA	NA	NA
LNCV6_128885_PI43004817	9.33397E-05	3.122296732	3.122296732	up	13.36966504	13.11539092	13.21414226	P	P	P	11.70726882	11.52299396	11.54547695	P	P	P	LNCV6_128885_F	mRNA	TCTAGGTTTTG	NM_021158	RefSeq	chr20	+	380628	397559	TRIB3	tribbles pseudokina	57761	GO:0008286|GO:0	.	.	-	.	NA	NA	NA	NA	NA	NA	NA	NA	NA	NA	NA	NA	NA
LNCV6_128387_PI43004817	2.58299E-05	2.370777918	2.370777918	up	13.28654556	13.23976466	13.29328814	P	P	P	11.96900427	12.05666797	12.05666797	P	P	P	LNCV6_128387_F	mRNA	CATGTTAATCA	NM_006636	RefSeq	chr2	+	74198562	74215297	MTHFD2	methylenetetrahyd	10797	GO:0000287|GO:0	.	.	-	.	NA	NA	NA	NA	NA	NA	NA	NA	NA	NA	NA	NA	NA
LNCV6_110769_PI43004817	0.000887425	2.644985725	2.644985725	up	8.80764692	8.762574496	8.567434469	P	P	P	7.084339103	7.420988302	7.409060776	P	P	P	LNCV6_110769_F	mRNA	CGTGGTATATC	NM_019593	RefSeq	chr20	-	5544433	5611026	GPCPD1	glycerophosphoche	56261	GO:0007519|GO:0	.	.	-	.	NA	NA	NA	NA	NA	NA	NA	NA	NA	NA	NA	NA	NA
LNCV6_128913_PI43004817	3.82674E-05	2.032505335	2.032505335	up	12.38119711	12.46768413	12.36846204	P	P	P	11.30775154	11.4226182	11.41636326	P	P	P	LNCV6_128913_F	mRNA	GGCATGTGCAC	NM_012234	RefSeq	chr3	-	72374592	72446623	RYBP	RING1 and YY1 bi	23429	GO:0031519|GO:0	.	.	-	.	NA	NA	NA	NA	NA	NA	NA	NA	NA	NA	NA	NA	NA
LNCV6_126944_PI43004817	0.000131168	2.72292943	2.72292943	up	9.382200197	9.408011619	9.279145122	P	P	P	7.787313611	7.989202885	7.952641861	P	P	P	LNCV6_126944_F	mRNA	CAGACATCTGG	NM_003620	RefSeq	chr17	+	60600182	60666279	PPM1D	protein phosphatas	8493	GO:0009314|GO:0	.	.	-	.	NA	NA	NA	NA	NA	NA	NA	NA	NA	NA	NA	NA	NA
LNCV6_134155_PI43004817	0.027876316	2.217914419	2.217914419	up	5.862864163	5.479389781	5.238714392	P	P	P	4.224640758	4.865536404	3.957010233	A	P	A	LNCV6_134155_F	mRNA	CCTGAGCCAAA	NM_015063	RefSeq	chr19	-	47428021	47472050	SLC8A2	solute carrier famil	6543	GO:0005516|GO:0	.	.	-	.	NA	NA	NA	NA	NA	NA	NA	NA	NA	NA	NA	NA	NA
LNCV6_127561_PI43004817	0.046471789	2.000136603	2.000136603	up	5.562934701	4.922499885	5.116057023	P	P	P	3.853330684	4.73388531	3.913880484	A	P	A	LNCV6_127561_F	mRNA	GCGAAAGATCA	NM_000236	RefSeq	chr15	+	58431975	58568874	LIPC	lipase%2C hepatic	3990	GO:0042632|GO:0	.	.	-	.	NA	NA	NA	NA	NA	NA	NA	NA	NA	NA	NA	NA	NA
LNCV6_129342_PI43004817	0.000599966	0.438825514	2.278810071	down	9.232470455	9.190500376	9.412948024	P	P	P	10.29070124	10.53039284	10.57368284	P	P	P	LNCV6_129342_F	mRNA	TTATTAGAGAT	NM_000609	RefSeq	chr10	-	44370152	44385097	CXCL12	chemokine (C-X-C	6387	GO:0008344|GO:0	.	.	-	.	NA	NA	NA	NA	NA	NA	NA	NA	NA	NA	NA	NA	NA
LNCV6_143196_PI43004817	0.000386538	2.008855059	2.008855059	up	10.51803674	10.64740791	10.49491757	P	P	P	9.420689274	9.578027718	9.638578155	P	P	P	LNCV6_143196_F	mRNA	GTGTCACTGTT	NM_031296	RefSeq	chr4	+	139453806	139475915	RAB33B	RAB33B%2C men	83452	GO:0005515|GO:0	.	.	-	.	NA	NA	NA	NA	NA	NA	NA	NA	NA	NA	NA	NA	NA
LNCV6_133264_PI43004817	0.001023811	3.516510411	3.516510411	up	9.738271572	9.706850475	9.659707889	P	P	P	7.745678357	7.876789191	8.027232582	P	P	P	LNCV6_133264_F	mRNA	CAGCATTCTAG	NM_177925	RefSeq	chr12	+	14774335	14778002	H2AFJ	H2A histone family	55766	GO:0008150|GO:0	.	.	-	.	NA	NA	NA	NA	NA	NA	NA	NA	NA	NA	NA	NA	NA
LNCV6_127635_PI43004817	5.60314E-05	17.08146453	17.08146453	up	6.321756554	6.545890705	6.736014793	P	P	P	2.234329506	2.316800021	2.745370228	A	A	A	LNCV6_127635_F	mRNA	CGAGAAGTCTG	NM_014553	RefSeq	chr2	-	121216587	121285202	TFCP2L1	transcription factor	29842	GO:0006694|GO:0	.	.	-	.	NA	NA	NA	NA	NA	NA	NA	NA	NA	NA	NA	NA	NA
LNCV6_139558_PI43004817	0.004520704	2.291748571	2.291748571	up	6.304052095	6.170966038	6.121613014	P	P	P	5.203143588	5.010526614	4.766979458	P	P	A	LNCV6_139558_F	mRNA	GAGTTGATGCA	NM_182971	RefSeq	chr14_KI270847v1	+	461752	462916	COX8C	cytochrome c oxid	NA	NA	.	.	-	.	NA	NA	NA	NA	NA	NA	NA	NA	NA	NA	NA	NA	NA
LNCV6_129237_PI43004817	0.039493497	2.4231826	2.4231826	up	5.086950028	5.05626682	5.117819763	P	P	P	3.427307592	3.538273459	4.300928944	A	A	A	LNCV6_129237_F	mRNA	CAAAAACAGCA	NM_001282417	RefSeq	chr17	+	18950675	19020691	SLC5A10	solute carrier famil	125206	GO:0005215|GO:0	.	.	-	.	NA	NA	NA	NA	NA	NA	NA	NA	NA	NA	NA	NA	NA
LNCV6_131572_PI43004817	1.62123E-05	2.581795774	2.581795774	up	8.829473703	8.741555411	8.82504988	P	P	P	7.40978734	7.503088437	7.376774836	P	P	P	LNCV6_131572_F	mRNA	CTGTCCTAAAG	NM_001184996	RefSeq	chr7	+	156640746	156677126	RNF32	ring finger protein	140545	GO:0005515|GO:0	.	.	-	.	NA	NA	NA	NA	NA	NA	NA	NA	NA	NA	NA	NA	NA
LNCV6_128757_PI43004817	0.000286392	3.02811069	3.02811069	up	5.800673191	5.969968797	5.697059809	P	P	P	4.37752209	4.04031515	4.247995723	A	A	A	LNCV6_128757_F	mRNA	CGGTGTGGGGA	NM_001657	RefSeq	chr4	+	74445097	74455009	AREG	amphiregulin	374	GO:0005515|GO:0	.	.	-	.	NA	NA	NA	NA	NA	NA	NA	NA	NA	NA	NA	NA	NA
LNCV6_129299_PI43004817	0.018878546	0.466603677	2.143146418	down	4.1132086	3.782796796	3.547428791	A	A	A	4.544567505	5.295414227	4.859746718	P	P	P	LNCV6_129299_F	mRNA	CCAGTGATGTT	NM_017594	RefSeq	chr9	-	90609831	90643105	DIRAS2	DIRAS family%2C	54769	GO:0006184|GO:0	.	.	-	.	NA	NA	NA	NA	NA	NA	NA	NA	NA	NA	NA	NA	NA
LNCV6_126937_PI43004817	0.000154477	2.460712094	2.460712094	up	8.125670472	8.173396814	8.313353871	P	P	P	6.961090399	6.843361887	6.914965482	P	P	P	LNCV6_126937_F	mRNA	CTTCAGCTTGG	NM_206539	RefSeq	chr6	-	43450351	43456632	DLK2	delta-like 2 homolo	65989	GO:0045746|GO:0	.	.	-	.	NA	NA	NA	NA	NA	NA	NA	NA	NA	NA	NA	NA	NA
LNCV6_58566_PI430048170	0.000181888	2.520483836	2.520483836	up	7.946158179	7.880448505	7.800929983	P	P	P	6.55365164	6.508060695	6.567709687	P	P	P	LNCV6_58566_PI	mRNA	AGCTGAGGCAG	NM_002286	RefSeq	chr12	+	6772503	6778455	LAG3	lymphocyte-activat	3902	GO:0045085|GO:0	.	.	-	.	NA	NA	NA	NA	NA	NA	NA	NA	NA	NA	NA	NA	NA
LNCV6_133527_PI43004817	0.000849155	5.245602217	5.245602217	up	7.932317524	7.950321237	7.905458511	P	P	P	5.379526182	5.64544478	5.577122985	P	P	P	LNCV6_133527_F	mRNA	GCTGTTTGTAG	NM_001010887	RefSeq	chr9	+	19408926	19452502	ACER2	alkaline ceramidase	NA	NA	.	.	-	.	NA	NA	NA	NA	NA	NA	NA	NA	NA	NA	NA	NA	NA
LNCV6_94130_PI430048170	0.000787557	0.46347184	2.157628389	down	8.650630784	8.918398311	8.718756384	P	P	P	10.04798357	9.789628409	9.775482356	P	P	P	LNCV6_94130_PI	mRNA	CTTGTAAATGA	NM_018136	RefSeq	chr1	-	197084126	197146694	ASPM	asp (abnormal spin	259266	GO:0097150|GO:0	.	.	-	.	NA	NA	NA	NA	NA	NA	NA	NA	NA	NA	NA	NA	NA
LNCV6_138526_PI43004817	0.000877371	2.420575265	2.420575265	up	9.069187745	8.766486119	9.054431664	P	P	P	7.484909876	7.751870609	7.8252129	P	P	P	LNCV6_138526_F	mRNA	AATGGTATCTG	NM_139314	RefSeq	chr19	+	8364126	8374375	ANGPTL4	angiopoietin-like 4	51129	GO:0004857|GO:0	.	.	-	.	NA	NA	NA	NA	NA	NA	NA	NA	NA	NA	NA	NA	NA
LNCV6_132659_PI43004817	0.001606198	2.428571958	2.428571958	up	8.258113958	7.98289359	7.8534206	P	P	P	6.675570311	6.677387049	6.917509728	P	P	P	LNCV6_132659_F	mRNA	TTACTGTGTAA	NM_004205	RefSeq	chr11	-	119355214	119381726	USP2	ubiquitin specific p	9099	GO:0005515|GO:0	.	.	-	.	NA	NA	NA	NA	NA	NA	NA	NA	NA	NA	NA	NA	NA
LNCV6_135408_PI43004817	0.006770474	0.441458871	2.265216684	down	6.036276091	6.103949093	5.584024745	P	P	P	6.99669003	7.019246924	7.282666367	P	P	P	LNCV6_135408_F	mRNA	CCTCAGCTCAG	NM_020776	RefSeq	chr18	+	36829116	37225325	KIAA1328	KIAA1328	57536	NA	.	.	-	.	NA	NA	NA	NA	NA	NA	NA	NA	NA	NA	NA	NA	NA
LNCV6_141610_PI43004817	0.001787012	8.882899077	8.882899077	up	6.009056324	5.660042781	5.934998931	P	P	P	2.944312429	2.208783176	2.908818799	A	A	A	LNCV6_141610_F	mRNA	GAGGTCCGTGT	NM_016540	RefSeq	chr11	-	94377310	94401419	GPR83	G protein-coupled r	10888	GO:0004983|GO:0	.	.	-	.	NA	NA	NA	NA	NA	NA	NA	NA	NA	NA	NA	NA	NA
LNCV6_142936_PI43004817	0.034133919	0.336881759	2.96840055	down	5.566426883	5.405197581	5.748945762	P	P	P	6.441620004	7.493333904	7.312746241	P	P	P	LNCV6_142936_F	mRNA	ATGTTCTGTGA	NM_007021	RefSeq	chr10	-	44976260	44978882	C10orf10	chromosome 10 op	11067	GO:0005739	.	.	-	.	NA	NA	NA	NA	NA	NA	NA	NA	NA	NA	NA	NA	NA
LNCV6_134523_PI43004817	0.014577958	2.356800733	2.356800733	up	6.910798608	6.702715023	6.84895223	P	P	P	5.190660158	5.698337355	5.800087967	P	P	P	LNCV6_134523_F	mRNA	AGTCACGCCGT	NM_005211	RefSeq	chr5	-	150053290	150113372	CSF1R	colony stimulating	1436	GO:0008284|GO:0	.	.	-	.	NA	NA	NA	NA	NA	NA	NA	NA	NA	NA	NA	NA	NA
LNCV6_139028_PI43004817	0.025064216	3.106034879	3.106034879	up	6.12338447	6.139856128	5.870336071	P	P	P	3.732904106	4.688961281	4.637580204	A	A	A	LNCV6_139028_F	mRNA	TGGGGCACACC	NM_005170	RefSeq	chr11	-	2268497	2270952	ASCL2	achaete-scute famil	430	GO:0001666|GO:0	.	.	-	.	NA	NA	NA	NA	NA	NA	NA	NA	NA	NA	NA	NA	NA
LNCV6_132238_PI43004817	0.000171811	2.209440333	2.209440333	up	10.08074632	10.01924558	10.22746372	P	P	P	8.902189879	8.923043326	9.073087027	P	P	P	LNCV6_132238_F	mRNA	CACGTTTGAAG	NM_005324	RefSeq	chr17	-	75776433	75779935	H3F3B	H3 histone%2C far	NA	NA	.	.	-	.	NA	NA	NA	NA	NA	NA	NA	NA	NA	NA	NA	NA	NA
LNCV6_133828_PI43004817	0.018141767	5.859321297	5.859321297	up	5.362497826	5.124501154	5.291401082	P	P	P	2.209924673	2.211502097	3.378617093	A	A	A	LNCV6_133828_F	mRNA	GAACTGGTCCG	NM_001886	RefSeq	chr22	+	26621963	26630672	CRYBA4	crystallin%2C beta	1413	GO:0003674|GO:0	.	.	-	.	NA	NA	NA	NA	NA	NA	NA	NA	NA	NA	NA	NA	NA
LNCV6_134509_PI43004817	0.005032321	9.663027001	9.663027001	up	7.030040637	6.277825007	6.431476321	P	P	P	4.038271844	2.91424374	2.704531497	A	A	A	LNCV6_134509_F	mRNA	GAGTGTTGCAA	NM_002438	RefSeq	chr10	+	17809342	17911162	MRC1	mannose receptor%	4360	GO:0005515|GO:0	.	.	-	.	NA	NA	NA	NA	NA	NA	NA	NA	NA	NA	NA	NA	NA
LNCV6_64654_PI430048170	0.036455789	2.357603155	2.357603155	up	6.998513522	7.221743062	7.994351551	P	P	P	6.636370713	6.062615726	5.892108329	P	P	P	LNCV6_64654_PI	mRNA	TACAGTTTTGA	NM_014686	RefSeq	chr19_KI270866v1	+	705	4760	KIAA0355	KIAA0355	9710	NA	.	.	-	.	NA	NA	NA	NA	NA	NA	NA	NA	NA	NA	NA	NA	NA
LNCV6_142033_PI43004817	0.000647801	0.475693515	2.102193889	down	9.313388717	9.563361701	9.35066704	P	P	P	10.32455636	10.53648748	10.5819994	P	P	P	LNCV6_142033_F	mRNA	AAAGCGAGTGT	NM_014033	RefSeq	chr12	+	50924750	50932517	METTL7A	methyltransferase l	25840	GO:0005811|GO:0	.	.	-	.	NA	NA	NA	NA	NA	NA	NA	NA	NA	NA	NA	NA	NA
LNCV6_127675_PI43004817	0.001220744	4.152461237	4.152461237	up	8.233858588	7.962572736	8.265485811	P	P	P	6.080919618	5.805176096	6.376234374	P	P	P	LNCV6_127675_F	mRNA	CCTAGAGTTCC	NM_013251	RefSeq	chr12	-	57009996	57016560	TAC3	tachykinin 3%2C tr	6866	GO:0005515|GO:0	.	.	-	.	NA	NA	NA	NA	NA	NA	NA	NA	NA	NA	NA	NA	NA
LNCV6_133178_PI43004817	0.000468875	2.420551774	2.420551774	up	7.233055749	7.288395324	7.117417481	P	P	P	6.073058062	5.79615238	5.935615638	P	P	P	LNCV6_133178_F	mRNA	GCTTCCCTTCA	NM_015460	RefSeq	chr3	+	39809659	40260320	MYRIP	myosin VIIA and R	25924	GO:0005515|GO:0	.	.	-	.	NA	NA	NA	NA	NA	NA	NA	NA	NA	NA	NA	NA	NA
LNCV6_134362_PI43004817	0.005193693	2.388109735	2.388109735	up	11.80131261	11.91793213	11.77485815	P	P	P	10.31459627	10.68663636	10.69739419	P	P	P	LNCV6_134362_F	mRNA	TTCAGGGAGGA	NM_012189	RefSeq	chr18	+	24138955	24161600	CABYR	calcium binding tyr	26256	GO:0017124|GO:0	.	.	-	.	NA	NA	NA	NA	NA	NA	NA	NA	NA	NA	NA	NA	NA
LNCV6_135627_PI43004817	0.000915708	2.408583617	2.408583617	up	7.538987513	7.682987095	7.48611916	P	P	P	6.296401944	6.339187398	6.274458294	P	P	P	LNCV6_135627_F	mRNA	GTACCATTGTC	NM_171998	RefSeq	chrX	-	155258233	155264589	RAB39B	RAB39B%2C men	116442	GO:0005515|GO:0	.	.	-	.	NA	NA	NA	NA	NA	NA	NA	NA	NA	NA	NA	NA	NA
LNCV6_128811_PI43004817	0.043045684	2.678636833	2.678636833	up	6.295945215	6.646557846	6.451156591	P	P	P	4.487255979	4.72729034	5.657727265	A	A	P	LNCV6_128811_F	mRNA	TCCTCTACTGC	NM_001258031	RefSeq	chr15	-	43794173	43800097	SERINC4	serine incorporator	619189	GO:0016021|GO:0	.	.	-	.	NA	NA	NA	NA	NA	NA	NA	NA	NA	NA	NA	NA	NA
LNCV6_136318_PI43004817	0.007383031	13.98092353	13.98092353	up	9.641569841	9.588415948	9.57507879	P	P	P	6.391337202	5.376291715	5.367085271	P	P	P	LNCV6_136318_F	mRNA	CCCGTAAAATC	NM_001301267	RefSeq	chr16	-	56666734	56668065	MT1G	metallothionein 1G	4495	GO:0071294|GO:0	.	.	-	.	NA	NA	NA	NA	NA	NA	NA	NA	NA	NA	NA	NA	NA
LNCV6_140943_PI43004817	9.01829E-05	8.224014227	8.224014227	up	6.917885212	6.539824081	6.640683295	P	P	P	3.505765112	3.930743802	3.528611615	A	A	A	LNCV6_140943_F	mRNA	GAGGCCAGTTC	NM_020530	RefSeq	chr22	-	30262829	30266840	OSM	oncostatin M	5008	GO:0042506|GO:0	.	.	-	.	NA	NA	NA	NA	NA	NA	NA	NA	NA	NA	NA	NA	NA
LNCV6_127538_PI43004817	0.000463389	3.099512117	3.099512117	up	6.844234674	6.91476143	6.845227896	P	P	P	5.255982999	5.330715902	5.114226108	P	P	P	LNCV6_127538_F	mRNA	TTCTGGGGCTT	NM_144676	RefSeq	chr16	-	69343245	69351809	TMED6	transmembrane em	146456	GO:0006810|GO:0	.	.	-	.	NA	NA	NA	NA	NA	NA	NA	NA	NA	NA	NA	NA	NA
LNCV6_139461_PI43004817	0.008207625	2.072847642	2.072847642	up	5.391024429	5.822352049	5.693232175	P	P	P	4.614632604	4.662141967	4.503716359	P	A	A	LNCV6_139461_F	mRNA	GGCTGTAAAGT	NM_000273	RefSeq	chrX	-	9725412	9765965	GPR143	G protein-coupled r	4935	GO:0005515|GO:0	.	.	-	.	NA	NA	NA	NA	NA	NA	NA	NA	NA	NA	NA	NA	NA
LNCV6_127781_PI43004817	8.16594E-05	2.22318302	2.22318302	up	11.50744226	11.34366774	11.48320579	P	P	P	10.2087871	10.34404785	10.3252691	P	P	P	LNCV6_127781_F	mRNA	GTACATCTTGT	NM_018645	RefSeq	chr2	-	238238266	238240124	HES6	hes family bHLH tr	55502	GO:0003712|GO:0	.	.	-	.	NA	NA	NA	NA	NA	NA	NA	NA	NA	NA	NA	NA	NA
LNCV6_132040_PI43004817	0.00814709	2.115095657	2.115095657	up	7.115962831	7.343873839	7.350916646	P	P	P	5.878790969	6.2723659	6.382490421	P	P	P	LNCV6_132040_F	mRNA	GTGTAAAGAAA	NM_001277403	RefSeq	chr19	+	23116974	23147212	ZNF730	zinc finger protein	100129543	GO:0006355|GO:0	.	.	-	.	NA	NA	NA	NA	NA	NA	NA	NA	NA	NA	NA	NA	NA
LNCV6_133837_PI43004817	0.002301069	5.008163508	5.008163508	up	9.906012839	9.977184456	9.921827799	P	P	P	7.41153189	7.548685787	7.839365793	P	P	P	LNCV6_133837_F	mRNA	TAAAGTCTTTA	NM_001080411	RefSeq	chr19	-	12014716	12035741	ZNF433	zinc finger protein	163059	GO:0006355|GO:0	.	.	-	.	NA	NA	NA	NA	NA	NA	NA	NA	NA	NA	NA	NA	NA
LNCV6_95119_PI430048170	0.001185621	2.09358627	2.09358627	up	13.67526554	13.40975689	13.55366809	P	P	P	12.53862804	12.50383228	12.40731916	P	P	P	LNCV6_95119_PI	mRNA	GCCTTATCTAA	NM_000270	RefSeq	chr14	+	20469378	20478006	PNP	purine nucleoside p	4860	GO:0046638|GO:0	.	.	-	.	NA	NA	NA	NA	NA	NA	NA	NA	NA	NA	NA	NA	NA
LNCV6_136744_PI43004817	0.008227132	2.171474159	2.171474159	up	7.036170132	7.189497116	7.479464884	P	P	P	6.055688647	6.122483121	6.202760918	P	P	P	LNCV6_136744_F	mRNA	CACCTATGCAC	NM_001243212	RefSeq	chr19	-	38908979	38912158	CCER2	coiled-coil glutama	643669	GO:0005576	.	.	-	.	NA	NA	NA	NA	NA	NA	NA	NA	NA	NA	NA	NA	NA
LNCV6_136413_PI43004817	0.006341564	2.358908415	2.358908415	up	5.782864315	5.72165706	5.641114485	P	P	P	4.469005507	4.689811375	4.241172439	P	A	A	LNCV6_136413_F	mRNA	CAAGTACCCTA	NM_152467	RefSeq	chr17	+	41837790	41848347	KLHL10	kelch-like family m	317719	GO:0009566|GO:0	.	.	-	.	NA	NA	NA	NA	NA	NA	NA	NA	NA	NA	NA	NA	NA
LNCV6_41248_PI430048170	0.003726655	2.224964285	2.224964285	up	6.483276211	6.485681128	6.810933152	P	P	P	5.368284344	5.447502828	5.523774999	P	P	P	LNCV6_41248_PI	mRNA	CAGAGCTGGAA	NM_001142569	RefSeq	chr1	+	200894820	200915736	C1orf106	chromosome 1 open	55765	NA	.	.	-	.	NA	NA	NA	NA	NA	NA	NA	NA	NA	NA	NA	NA	NA
LNCV6_135467_PI43004817	0.00017396	3.277947726	3.277947726	up	12.64766854	12.54280038	12.48252673	P	P	P	10.70369554	10.84870678	10.97439607	P	P	P	LNCV6_135467_F	mRNA	TGACTTCTCTG	NM_001190468	RefSeq	chr5	-	37812676	37835827	GDNF	glial cell derived ne	2668	GO:0001656|GO:0	.	.	-	.	NA	NA	NA	NA	NA	NA	NA	NA	NA	NA	NA	NA	NA
LNCV6_142339_PI43004817	0.024989871	3.308185249	3.308185249	up	6.227932155	6.467985899	6.150121833	P	P	P	4.047920329	4.265524411	5.133807694	A	A	P	LNCV6_142339_F	mRNA	GGCTAGAAAAG	NM_018423	RefSeq	chr12	-	10618938	10674292	STYK1	serine/threonine/ty	55359	GO:0005515|GO:0	.	.	-	.	NA	NA	NA	NA	NA	NA	NA	NA	NA	NA	NA	NA	NA
LNCV6_143052_PI43004817	0.017122969	0.469192893	2.131319581	down	4.503264872	4.913161407	4.838171735	P	P	P	5.417501895	6.078314686	5.982063525	P	P	P	LNCV6_143052_F	mRNA	GAAGTGCTCTT	NM_173485	RefSeq	chr20	+	52972406	53495330	TSHZ2	teashirt zinc finger	128553	GO:0006355|GO:0	.	.	-	.	NA	NA	NA	NA	NA	NA	NA	NA	NA	NA	NA	NA	NA
LNCV6_135446_PI43004817	0.00062878	0.477629685	2.093672214	down	9.774468347	9.866885214	9.934612992	P	P	P	10.95254798	10.89461961	10.93077885	P	P	P	LNCV6_135446_F	mRNA	GTGTAATCTAC	NM_001166691	RefSeq	chr6	+	80004604	80042527	TTK	TTK protein kinase	7272	GO:0005515|GO:0	.	.	-	.	NA	NA	NA	NA	NA	NA	NA	NA	NA	NA	NA	NA	NA
LNCV6_135795_PI43004817	0.009083358	7.142742308	7.142742308	up	7.132503328	7.071748192	7.239213759	P	P	P	3.767903876	4.184082684	4.798594146	A	A	A	LNCV6_135795_F	mRNA	CTCATGCTTGC	NM_004816	RefSeq	chr9	+	69329324	69392454	FAM189A2	family with sequen	9413	GO:0008150|GO:0	.	.	-	.	NA	NA	NA	NA	NA	NA	NA	NA	NA	NA	NA	NA	NA
LNCV6_142761_PI43004817	0.000128018	4.610860797	4.610860797	up	10.25265538	10.20989805	10.12118528	P	P	P	7.857983172	7.98289359	8.119031195	P	P	P	LNCV6_142761_F	mRNA	CAAATTTGGAT	NM_001969	RefSeq	chr14	+	103334001	103345024	EIF5	eukaryotic translati	1983	GO:0010467|GO:0	.	.	-	.	NA	NA	NA	NA	NA	NA	NA	NA	NA	NA	NA	NA	NA
LNCV6_135639_PI43004817	0.005219629	7.715324642	7.715324642	up	5.786826818	5.924024922	5.681309215	P	P	P	2.336199014	3.258674944	2.818786903	A	A	A	LNCV6_135639_F	mRNA	CTCTTTCTCTGC	NM_207007	RefSeq	chr17_KI270909v1	+	165044	167015	CCL4L1	chemokine (C-C m	NA	NA	.	.	-	.	NA	NA	NA	NA	NA	NA	NA	NA	NA	NA	NA	NA	NA
LNCV6_138176_PI43004817	0.010392935	3.85595277	3.85595277	up	7.736125994	6.70377542	7.293094085	P	P	P	5.784656742	4.73151355	5.3683677	P	A	P	LNCV6_138176_F	mRNA	CTGTGTTGAAT	NM_000584	RefSeq	chr4	+	73740505	73743716	CXCL8	chemokine (C-X-C	3576	GO:0005515|GO:0	.	.	-	.	NA	NA	NA	NA	NA	NA	NA	NA	NA	NA	NA	NA	NA
LNCV6_141446_PI43004817	0.005929246	2.535800832	2.535800832	up	6.4225996	6.282040123	6.388405569	P	P	P	5.211645506	4.752440505	5.06788068	P	P	P	LNCV6_141446_F	mRNA	GTTGCCACCTG	NM_000596	RefSeq	chr7	+	45888359	45893668	IGFBP1	insulin-like growth	3484	GO:0008286|GO:0	.	.	-	.	NA	NA	NA	NA	NA	NA	NA	NA	NA	NA	NA	NA	NA
LNCV6_128936_PI43004817	0.009891287	2.159655285	2.159655285	up	7.977951498	8.010747862	8.013576897	P	P	P	6.65757408	7.042322584	6.94316748	P	P	P	LNCV6_128936_F	mRNA	AGCGAGCACAT	NM_021147	RefSeq	chr5	-	55231152	55233717	CCNO	cyclin O%2C trans	10309	GO:0004844|GO:0	.	.	-	.	NA	NA	NA	NA	NA	NA	NA	NA	NA	NA	NA	NA	NA
LNCV6_139291_PI43004817	0.002122524	3.042548435	3.042548435	up	7.123981689	7.046230747	7.138840744	P	P	P	5.547858095	5.629561303	5.297077058	P	P	P	LNCV6_139291_F	mRNA	GAACACTTTCA	NM_017413	RefSeq	chrX	-	129645258	129654956	APLN	apelin	8862	GO:0007165|GO:0	.	.	-	.	NA	NA	NA	NA	NA	NA	NA	NA	NA	NA	NA	NA	NA
LNCV6_137012_PI43004817	0.000210561	3.034961276	3.034961276	up	5.829045979	6.115961722	5.898131386	P	P	P	4.223980186	4.407938798	4.413987331	A	A	A	LNCV6_137012_F	mRNA	AGACATTGGCC	NM_207627	RefSeq	chr21	+	42199688	42297244	ABCG1	ATP-binding casse	9619	GO:0005515|GO:0	.	.	-	.	NA	NA	NA	NA	NA	NA	NA	NA	NA	NA	NA	NA	NA
LNCV6_65687_PI430048170	0.000455944	0.390461898	2.561069352	down	8.325783201	8.43065549	8.425610711	P	P	P	9.611181665	9.825463251	9.808376308	P	P	P	LNCV6_65687_PI	mRNA	TCCTTTCAGTG	NM_138370	RefSeq	chr2	+	42048020	42058528	PKDCC	protein kinase dom	91461	GO:0005794|GO:0	.	.	-	.	NA	NA	NA	NA	NA	NA	NA	NA	NA	NA	NA	NA	NA
LNCV6_127609_PI43004817	0.001056823	3.173826166	3.173826166	up	6.853386622	6.674663931	6.532660005	P	P	P	4.861721511	4.888171333	5.289186668	P	P	P	LNCV6_127609_F	mRNA	GACCAGGAAAT	NM_020683	RefSeq	chr1	-	111483347	111504121	TMIGD3	transmembrane and	57413	GO:0005886|GO:0	.	.	-	.	NA	NA	NA	NA	NA	NA	NA	NA	NA	NA	NA	NA	NA
LNCV6_140973_PI43004817	0.001443635	3.317902802	3.317902802	up	5.751122949	6.133892065	6.074359287	P	P	P	3.974441053	4.231669587	4.536148869	A	A	A	LNCV6_140973_F	mRNA	CAGCTTTGGCA	NM_173076	RefSeq	chr2	-	214931541	215138428	ABCA12	ATP-binding casse	26154	GO:0005515|GO:0	.	.	-	.	NA	NA	NA	NA	NA	NA	NA	NA	NA	NA	NA	NA	NA
LNCV6_129050_PI43004817	0.005272976	2.107640653	2.107640653	up	6.062615726	5.88810203	5.902783815	P	P	P	4.630590398	5.045172964	4.926478734	P	P	P	LNCV6_129050_F	mRNA	TGACTGTTTCC	NM_006186	RefSeq	chr2	-	156324431	156332775	NR4A2	nuclear receptor su	4929	GO:0005515|GO:0	.	.	-	.	NA	NA	NA	NA	NA	NA	NA	NA	NA	NA	NA	NA	NA
LNCV6_144934_PI43004817	0.003981201	3.398740322	3.398740322	up	7.21414521	7.133579451	6.977663255	P	P	P	4.993708021	5.564216827	5.423206245	P	P	P	LNCV6_144934_F	mRNA	TCGCTACAGTA	NM_022135	RefSeq	chr3	-	119642051	119660590	POPDC2	popeye domain con	64091	GO:0008150|GO:0	.	.	-	.	NA	NA	NA	NA	NA	NA	NA	NA	NA	NA	NA	NA	NA
LNCV6_129680_PI43004817	0.014300155	2.084066934	2.084066934	up	6.757342958	6.431300663	6.335846764	P	P	P	5.073562627	5.746881974	5.482307386	P	P	P	LNCV6_129680_F	mRNA	CCTATAAGACA	NM_002098	RefSeq	chr6	-	42183283	42194956	GUCA1B	guanylate cyclase a	2979	GO:0007589|GO:0	.	.	-	.	NA	NA	NA	NA	NA	NA	NA	NA	NA	NA	NA	NA	NA
LNCV6_123979_PI43004817	0.031387478	0.402018732	2.487446281	down	6.347093236	6.438095005	6.485346478	P	P	P	7.248348871	7.770668517	8.080133717	P	P	P	LNCV6_123979_F	mRNA	GCAAACGCTGG	NM_003107	RefSeq	chr6	+	21593740	21598618	SOX4	SRY (sex determin	6659	GO:0005515|GO:0	.	.	-	.	NA	NA	NA	NA	NA	NA	NA	NA	NA	NA	NA	NA	NA
LNCV6_134164_PI43004817	0.000780538	0.347302746	2.879332257	down	7.689538425	7.897222069	7.836043936	P	P	P	9.121351982	9.466847393	9.396997224	P	P	P	LNCV6_134164_F	mRNA	GCATTGGAGAA	NM_001290268	RefSeq	chr20	-	50586109	50691530	FAM65C	family with sequen	140876	NA	.	.	-	.	NA	NA	NA	NA	NA	NA	NA	NA	NA	NA	NA	NA	NA
LNCV6_136521_PI43004817	0.008907377	3.806010256	3.806010256	up	5.587083156	5.876504586	5.622902734	P	P	P	3.275131092	3.787110622	4.131598127	A	A	A	LNCV6_136521_F	mRNA	CTAGATTGGAT	NM_001252100	RefSeq	chr1	-	200969385	201023700	KIF21B	kinesin family mem	23046	GO:0008017|GO:0	.	.	-	.	NA	NA	NA	NA	NA	NA	NA	NA	NA	NA	NA	NA	NA
LNCV6_140856_PI43004817	0.001654197	2.185445801	2.185445801	up	11.13770982	11.19514066	11.1260192	P	P	P	9.891492427	10.06402748	10.11135906	P	P	P	LNCV6_140856_F	mRNA	AGGGGCAAAAC	NM_014771	RefSeq	chr16	+	30761611	30776307	RNF40	ring finger protein	9810	GO:0005515|GO:0	.	.	-	.	NA	NA	NA	NA	NA	NA	NA	NA	NA	NA	NA	NA	NA
LNCV6_131124_PI43004817	0.018991495	2.606833711	2.606833711	up	7.492947393	7.188268541	7.127998275	P	P	P	5.363531647	6.246490431	5.945104768	P	P	P	LNCV6_131124_F	mRNA	GGGTCACAGAG	NM_003890	RefSeq	chr19	-	39863322	39934626	FCGBP	Fc fragment of IgG	8857	GO:0005515|GO:0	.	.	-	.	NA	NA	NA	NA	NA	NA	NA	NA	NA	NA	NA	NA	NA
LNCV6_137556_PI43004817	0.000229064	0.409675801	2.440954526	down	10.10314301	10.27990884	10.35460441	P	P	P	11.42373818	11.63217709	11.54791922	P	P	P	LNCV6_137556_F	mRNA	CCTACCTTTGT	NM_001001418	RefSeq	chr17_KI270909v1	-	207819	218757	TBC1D3C	TBC1 domain fami	414060	GO:0005886|GO:0	.	.	-	.	NA	NA	NA	NA	NA	NA	NA	NA	NA	NA	NA	NA	NA
LNCV6_138714_PI43004817	0.004804849	4.123640713	4.123640713	up	4.286793194	5.023087058	5.07537974	P	P	P	2.794730989	3.168429541	2.282555453	A	A	A	LNCV6_138714_F	mRNA	GGAGGCTTTTG	NM_001039775	RefSeq	chr1	-	26321858	26354130	AIM1L	absent in melanom	55057	GO:0030246	.	.	-	.	NA	NA	NA	NA	NA	NA	NA	NA	NA	NA	NA	NA	NA
LNCV6_139555_PI43004817	0.000831164	2.024999428	2.024999428	up	6.070639488	5.95557641	5.796649361	P	P	P	4.773873019	4.947876593	5.047244341	P	P	P	LNCV6_139555_F	mRNA	CATGGCTACCC	NM_001278082	RefSeq	chr5	-	151769792	151771508	LOC100652758	uncharacterized LC	NA	NA	.	.	-	.	NA	NA	NA	NA	NA	NA	NA	NA	NA	NA	NA	NA	NA
LNCV6_131010_PI43004817	8.63694E-05	5.714839895	5.714839895	up	10.03555869	9.776875397	9.761944097	P	P	P	7.246379899	7.424097127	7.370764988	P	P	P	LNCV6_131010_F	mRNA	GAGTCAGAAAT	NM_032464	RefSeq	chr7	+	74209756	74229834	LAT2	linker for activatio	7462	GO:0005515|GO:0	.	.	-	.	NA	NA	NA	NA	NA	NA	NA	NA	NA	NA	NA	NA	NA
LNCV6_127777_PI43004817	0.002745474	2.568213182	2.568213182	up	5.605969099	6.047771934	5.826130448	P	P	P	4.247328924	4.372491666	4.760666729	A	A	A	LNCV6_127777_F	mRNA	AGTGAGGATAA	NM_019089	RefSeq	chr1	-	6415233	6419919	HES2	hes family bHLH tr	54626	GO:0003690|GO:0	.	.	-	.	NA	NA	NA	NA	NA	NA	NA	NA	NA	NA	NA	NA	NA
LNCV6_138865_PI43004817	0.003920916	6.540188856	6.540188856	up	10.71562215	10.74510537	10.67157836	P	P	P	7.63985016	8.044430368	8.254940022	P	P	P	LNCV6_138865_F	mRNA	GTTCTCTTCTTT	NM_004364	RefSeq	chr19	-	33299933	33302564	CEBPA	CCAAT/enhancer	1050	GO:0005515|GO:0	.	.	-	.	NA	NA	NA	NA	NA	NA	NA	NA	NA	NA	NA	NA	NA
LNCV6_80106_PI430048170	0.007631777	0.470728896	2.124365018	down	7.599563405	7.740800991	7.740586831	P	P	P	8.521456948	8.946835965	8.844952861	P	P	P	LNCV6_80106_PI	mRNA	TACAGACCATG	NM_001286841	RefSeq	chr8	+	98944402	98952104	OSR2	odd-skipped related	116039	GO:0001656|GO:0	.	.	-	.	NA	NA	NA	NA	NA	NA	NA	NA	NA	NA	NA	NA	NA
LNCV6_135156_PI43004817	0.017164415	3.70710404	3.70710404	up	6.389047418	6.188653623	6.40488533	P	P	P	3.839643376	4.828142325	4.48658996	A	P	A	LNCV6_135156_F	mRNA	GGGGGGAGGTT	NM_022467	RefSeq	chr19	+	33684527	33773509	CHST8	carbohydrate (N-ac	64377	GO:0016051|GO:0	.	.	-	.	NA	NA	NA	NA	NA	NA	NA	NA	NA	NA	NA	NA	NA
LNCV6_136507_PI43004817	0.002549685	2.388819001	2.388819001	up	5.822305391	5.974688112	5.79849958	P	P	P	4.780561201	4.636605771	4.389009633	P	A	A	LNCV6_136507_F	mRNA	GGGCCGTGGTA	NM_015163	RefSeq	chr14	-	50975262	51095704	TRIM9	tripartite motif con	114088	GO:0004842|GO:0	.	.	-	.	NA	NA	NA	NA	NA	NA	NA	NA	NA	NA	NA	NA	NA
