# Supplementary material for: Oxidative Stress-Induced circHBEGF Promotes Extracellular Matrix Production via Regulating miR-646/EGFR in Human Trabecular Meshwork Cells
Source: Oxid Med Cell Longev. 2020 Nov 24;2020:4692034. doi: 10.1155/2020/4692034 (PMC7722639; doi:10.1155/2020/4692034)
Supplement: Supplementary 5 — Table S3: the sequences of the RT-PCR primers (5′-3′). [file 4692034.f5.pdf]

Table S3. The sequences of the RT-PCR primers (5' - 3')	
hsa_circ_0080940-F	CTGTAGGAACAGGCATAGGAGAT
hsa_circ_0080940-R	AGCCAAGATGATGCAGCGTTC
hsa_circ_0080942-F	GACTCCTCCCAGGCAATTTCT
hsa_circ_0080942-R	CTCAACATAGGTTCTAAGGATCCTC
hsa_circ_0074241-F	AAGTCACGGACTTTCCGGTC
hsa_circ_0074241-R	AGAGGCAGATCTGGACCTTTTG
hsa_circ_0009510-F	AGGTAGCTGTCGCAAGGCA
hsa_circ_0009510-R	CAGGGAACGTCTGGGAGAAC
hsa_circ_0050104-F	AGGAGCATCTGAGAGCCATTC
hsa_circ_0050104-R	CAGTTGTCCTGCCCTGTGGA
hsa_circ_0006895-F	CCTGCACCCACTCTTACTCA
hsa_circ_0006895-R	TGAGGAGGTCTGACTTGCTGA
hsa_circ_0080943-F	ACTGAGACCAGGTTGAGCTG
hsa_circ_0080943-R	AACAGCCTCCTTCACAAGGC
hsa_circ_0047835-F	TTGATGAAACGTGCACCTCCTG
hsa_circ_0047835-R	CAGCCTAGAGGCAGCTATTTTACC
hsa_circ_0074150-F	TCATGACTAAGGCCATTCCAGC
hsa_circ_0074150-R	CCTCATCTGTGCAGACTGACATC
hsa_circ_0031462-F	CACCATGCCGTAACAGAGTCTT
hsa_circ_0031462-R	TGGTCTATAGAGCATGTGGAGC
hsa_circ_0050202-F	AGCTGGTGTGCAGGAGGAA
hsa_circ_0050202-R	CAAAGCCCATTGCACGGGA
miR-4664-3p stem loop	GTCGTATCCAGTGCAGGGTCCGAGGTATTCGCACTG GATACGACCTGCCC
miR-4664-3p-F	GGTCCGAAGGCCAGACACTCG
miR-4687-5p stem loop	GTCGTATCCAGTGCAGGGTCCGAGGTATTCGCACTG GATACGACAAACCC
miR-4687-5p-F	GGTCCGTCGGGAGGAGGGCGT
miR-149-5p stem loop	GTCGTATCCAGTGCAGGGTCCGAGGTATTCGCACTG GATACGACCCCTCA
miR-149-5p-F	GGTCCAGACCGAGGCACAGAAG
miR-6131 stem loop	GTCGTATCCAGTGCAGGGTCCGAGGTATTCGCACTG GATACGACGTGAGG
miR-6131-F	GGTCCCCGACCAGTCTAC
miR-646 stem loop	GTCGTATCCAGTGCAGGGTCCGAGGTATTCGCACTG
miR-646-F	GGTCCTTCGTCGACGGAG
Universal reverse	GTGCAGGGTCCGAGGT
U6 stem loop	GTCGTATCCAGTGCAGGGTCCGAGGTATTCGCACTG GATACGACAAAATA
U6-F	CTCGCTTCGGCAGCACA

U6-R	AACGCTTCACGAATTTGCGT
Fibronectin-F	GAGAATAAGCTGTACCATCGCAA
Fibronectin-R	CGACCACATAGGAAGTCCCAG
Collagen I -F	ATCAACCGGAGGAATTTCCGT
Collagen I -R	CACCAGGACGACCAGGTTTTC
NOB1-F	CAGGACATCGGGAAGAACATTT
NOB1-R	CCGCACGTATTCCGGTAAGG
FGF2-F	AGAAGAGCGACCCTCACATCA
FGF2-R	CGGTTAGCACACACTCCTTTG
EGFR-F	AGGCACGAGTAACAAGCTCAC
EGFR-R	ATGAGGACATAACCAGCCACC
FOXK1-F	CATTACCCCTACTACCGGACG
FOXK1-R	GTAACGGTTCAAAGAGAGGTTGT
NPM1-F	GCACTTAGTAGCTGTGGAGGA
NPM1-R	TTCACTGGCGCTTTTTCTTCA
MIIP-F	ATACCTGGGCTATGACTGGATT
MIIP-R	AGTACACGCATTCATGGTCTTC
IGF1-F	GCTCTTCAGTTCGTGTGTGGA
IGF1-R	GCCTCCTTAGATCACAGCTCC
CDK6-F	CCAGATGGCTCTAACCTCAGT
CDK6-R	AACTTCCACGAAAAAGAGGCTT
